# Global, regional and national burden of myocarditis in adolescents and young adults, 1990–2021: systematic analysis of the global burden of disease study 2021

**DOI:** 10.3389/fcvm.2026.1623833

**Published:** 2026-03-26

**Authors:** Le Zhao, Yan Lou, Qisheng Gao

**Affiliations:** 1 Department of Nursing, Hangzhou Normal University, Hangzhou, Zhejiang, China; 2 School of Public Health, Hangzhou Medical College, Hangzhou, Zhejiang, China

**Keywords:** disability-adjusted life years, epidemiology, global burden of disease, incidence, myocarditis, social-demographic index

## Abstract

**Objective:**

This study aims to systematically characterize the global, regional, and national trends of myocarditis among individuals aged 15–39 years between 1990 and 2021, identify associated influencing factors, and forecast future burden through 2050. Findings from this research are expected to support the development of evidence-based health policies and enhance resource allocation for this vulnerable population.

**Methods:**

Data on incidence, mortality, and disability-adjusted life years (DALYs) attributable to myocarditis among individuals aged 15–39 years were obtained from the Global Burden of Disease (GBD) study. Analyses were stratified by region, country, age, sex, and sociodemographic index (SDI) to explore heterogeneity across populations. Temporal trends were evaluated by estimating the annual percentage change (APC) using the Segment regression model, with the estimated average annual percentage change (EAPC) calculated through log-linear models for robustness.

**Results:**

There were 349,033.33 incident cases of myocarditis in adolescents and young adults worldwide in 2021[95% uncertainty interval (UI), 235,755.91–494,002.44]. Mortality was recorded at 3,300.73 deaths (95% UI, 2,643.01–4,256.11), while DALYs totaled 208,644.28 (95% UI, 169,653.92–267,518.34). The high SDI region recorded the highest incidence rate among the five SDI categories, reaching 13.66 per 100,000 population (95% UI, 9.84–18.62). The greatest number of myocarditis-related deaths occurred regionally in East Asia (1,097.59; 95% UI, 726.99–1,426.58). India reported the most incident cases at the national level (71,257.12; 95% UI, 46,817.01–100,807.05). In 2021, the incidence, mortality, and DALYs of myocarditis were all higher in men than in women. The highest incidence was observed in the 30–34 years age group, whereas the 35–39 years age group had the highest mortality and DALYs. Both high and low temperatures were found to be significant risk factors for myocarditis in this group, based on the 2021 GBD data. Frontier analyses indicate that there is potential for alleviating the burden of myocarditis in adolescents and young adults, considering national and regional levels of development. Through 2050, incidence and DALYs rates are expected to continue to drop.

**Conclusion:**

While global incidence, mortality, and DALYs rates of myocarditis among adolescents and young adults have shown a declining trend, the numbers of incident cases, deaths, and DALYs continue to rise. Enhancing the understanding of its epidemiological characteristics can facilitate more effective prevention and control strategies.

## Introduction

Myocarditis, also known as inflammatory cardiomyopathy, is characterized by the presence of inflammatory cell infiltration and non-ischemic myocardial necrosis within the heart muscle, leading to structural damage and impaired cardiac function (1). While the majority of individuals with myocarditis experience favorable outcomes, a subset may develop severe complications such as dilated cardiomyopathy, cardiogenic shock, or sudden cardiac death, all of which significantly reduce quality of life and place a considerable strain on healthcare systems and society at large (2, 3). Globally, the estimated incidence of myocarditis ranged between 13.11 and 19.76 cases per 100,000 population, with mortality rising from 21,765 deaths in 1990 to 31,765 in 2021, indicating a steadily increasing trend (4). Myocarditis, a primary cause of sudden cardiac death, has become a significant global public health problem, especially for adolescents and young adults (5). Evidence suggests that up to 20% of patients younger than 40 may succumb to abrupt cardiac arrhythmias or cardiogenic shock (6). Notably, individuals aged 15–39 are considered a high-risk population, as the disease often presents abruptly and with minimal symptoms, making timely diagnosis and treatment difficult. This diagnostic delay can result in severe or even fatal outcomes (7). To mitigate this public health burden, sustained epidemiological monitoring of myocarditis among this vulnerable group is essential.

The Global Burden of Disease (GBD) 2021 study offers comprehensive data on diseases and risk factors across global, regional, and national levels, thus providing an essential scientific basis for the development of public health strategies and the more effective allocation of healthcare resources (8). Although previous GBD research has examined myocarditis across all age groups and focused to some extent on the 0–14-year-olds population (4, 9), there remains a lack of systematic investigation targeting the 15–39 age group in terms of global incidence, mortality, and disability-adjusted life years (DALYs). In addition to having a higher risk of myocarditis-related death, this demographic of people is also at an important stage in familial and societal development. Based on this, this study presents the first global analysis of the burden of myocarditis and its associated risk factors among individuals aged 15 to 39 years from 1990 to 2021, utilizing data from the GBD database, and projects trends in this population from 2022 to 2050. Moreover, it is the first study to incorporate annual percentage changes into the analysis of the global burden of myocarditis, enabling a more detailed assessment of trend variations over specific periods. Additionally, frontier analysis is employed for the first time to investigate the association between sociodemographic development levels and the burden of myocarditis-related DALYs among adolescents and young adults. The ultimate objective is to offer evidence-based backing for the creation of focused preventive, diagnosis, and treatment plans meant to lessen the global health burden in this susceptible group.

## Methods

### Overview and data collection

From 1990 to 2021, the GBD 2021 project conducted a thorough assessment of 369 illnesses and injuries in 204 nations and territories, with a primary focus on important health indicators including incidence, mortality, and DALYs (10). The research, which was overseen by the University of Washington's Institute for Health Metrics and Evaluation (IHME), sought to measure the effects that different illnesses, injuries, and risk factors had on health. GBD 2021 integrates diverse data sources, including national health departments, research institutions, universities, healthcare organizations, and health service providers, as well as population censuses and health interview surveys. Data on myocarditis cases, incidence, mortality, and DALYs in adolescents and young adults at the national, regional, and worldwide levels were collected for this study, along with the associated rates and uncertainty intervals. It also gathered information on worldwide risk factors that contribute to DALYs and mortality from myocarditis in adolescents and young adults. This study divided individuals into five age groups: 15–19, 20–24, 25–29, 30–34, and 35–39, in order to investigate the age distribution of the myocarditis burden among adolescents and young adults. Due to the absence of race and ethnicity data for participants in the GBD database, analyses involving these variables could not be performed. All data came from the GBD database (https://vizhub.healthdata.org/gbd-results/), which has data on 204 countries and territories (11). The Strengthening the Reporting of Observational Studies in Epidemiology (STROBE) normative principles were followed in this study (12).

### Sociodemographic index

A composite metric called the Social Demographic Index (SDI) is used to assess the socioeconomic development level of a nation or region and how it affects health. Three primary factors are used in its calculation: total fertility rate, which represents social development; average years of education, which indicates educational achievement; and per capita income, which represents economic resources (13). A higher level of socioeconomic development is indicated by higher SDI values, which range from 0 to 1. SDI categorizes nations and areas into five groups based on the GBD database: low (0.0–0.4), low-middle (0.4–0.6), middle (0.6–0.7), high-middle (0.7–0.8), and high (0.8–1.0). This categorization facilitates a comprehensive analysis of the impact of socioeconomic factors and regional differences on the myocarditis burden among adolescents and young adults.

### Frontier analysis

To examine the relationship between sociodemographic development and myocarditis burden in adolescents and young adults, we conducted a frontier analysis based on age-standardized DALYs rates and the Socio-demographic Index (SDI) (14). In this framework, the frontier represents the lowest achievable burden at a given SDI level, serving as a benchmark of optimal performance.

First, an empirical frontier was constructed as the lower envelope of observed DALYs rates along increasing SDI using a running-minimum procedure. We then applied locally weighted regression (LOESS) to obtain a smooth continuous frontier curve. The LOESS smoothing parameter (span) was selected using a bootstrap out-of-bag (OOB) procedure by comparing prediction errors across candidate spans (0.3, 0.4, and 0.5), representing low-to-moderate-to-higher smoothing levels.

To assess robustness, we refitted the frontier under each span and evaluated the stability of both the frontier shape and the country-level efficiency gaps. The efficiency gap (effective difference) was defined as the absolute deviation between a country's observed DALYs rate and the corresponding frontier value at the same SDI level in 2021 (eff_diff = observed−frontier), where larger values indicate greater potential for improvement. Bootstrap resampling was further used to quantify uncertainty around the estimated frontier curve. Detailed span-selection results and sensitivity analyses are reported in the Supplementary Material 1.

### Statistical analysis

Key metrics used to evaluate the burden of myocarditis among adolescents and young adults include incidence, mortality, and DALYs, as well as their respective rates. As reported in the GBD database, these rates are presented per 100,000 individuals and include a corresponding 95% UI (15). These 95% UIs were derived from 1,000 posterior draws generated within the GBD 2021 modeling framework, with the 2.5th and 97.5th percentiles taken as the lower and upper bounds, reflecting uncertainty arising from data availability, data quality, and model-based assumptions. We computed the annual percentage change (APC) and associated 95% confidence interval (CI) using the Segment regression model in order to examine trends over various periods. This approach facilitates the precise identification of inter-annual fluctuations and offers a comprehensive view of temporal trends (16). Joinpoints in the segmented regression were selected using a BIC-based model selection procedure (0 to Kmax joinpoints; Kmax = 5) implemented in the R package segmented (function selgmented, type = ‘bic'). APCs for each segment were derived from the segment-specific slopes on the log scale {APC = [exp(*β*)−1] × 100%}, with 95% CIs calculated accordingly. Model assumptions and adequacy were evaluated using residual diagnostics (residuals vs. fitted values and Q–Q plots) and independence checks (ACF/PACF of residuals and Durbin–Watson and Ljung–Box tests). Robustness was examined through sensitivity analyses by varying the number of joinpoints and using multiple alternative initial breakpoint sets; detailed diagnostic plots and sensitivity results are provided in the Supplementary Material 2. In order to investigate long-term trend patterns, we also used a log-linear regression model to estimate the average annual percentage change (EAPC) and its 95% CI for the incidence, mortality, and DALYs of myocarditis among adolescents and young adults from 1990 to 2021 (17). The EAPC indicates the direction of long-term trends, revealing whether a particular indicator is on the rise or decline. A significantly decreasing trend in an indicator is suggested when the upper bound of the 95% CI for the EAPC is negative. Conversely, a significantly increasing trend is indicated when the lower bound of the 95% CI is positive. We also fitted curve models to examine the association between disease burden and the SDI. The main risk factors for myocarditis in adolescents and young adults were also investigated in this study. In forecasting analyses, Bayesian age–period–cohort (BAPC) models were fitted using the R packages BAPC and INLA. Age effects were modeled as a second-order random walk (RW2), period effects as a first-order random walk (RW1), and cohort effects as RW2. Log-gamma priors were assigned to the precision parameters of the structured effects (shape = 1, rate = 0.00005), and an additional i.i.d. overdispersion component was included to account for extra-Poisson variability with a log-gamma prior (shape = 1, rate = 0.005). Age-standardized rates were computed using WHO standard population weights. Model fitting in INLA is based on deterministic approximation rather than MCMC sampling; therefore, model adequacy was assessed using retrospective validation (retro = TRUE) and predictive accuracy metrics. Specifically, observed vs. fitted age-standardized rates during 1990–2021 were compared, and goodness-of-fit was summarized using RMSE, MAE, and empirical coverage of the 95% predictive intervals (18). The detailed specifics of the BAPC model are provided in Supplementary Material 3. R software (version 4.4.3) and the JD_GBDR platform (version V2.37, Jingding Medical Technology Co., Ltd.) were used for all data analysis and visualization activities in this work, with a *p* < 0.05 criterion for statistical significance.

## Results

### Global level

#### Incidence

**Figure 1:**
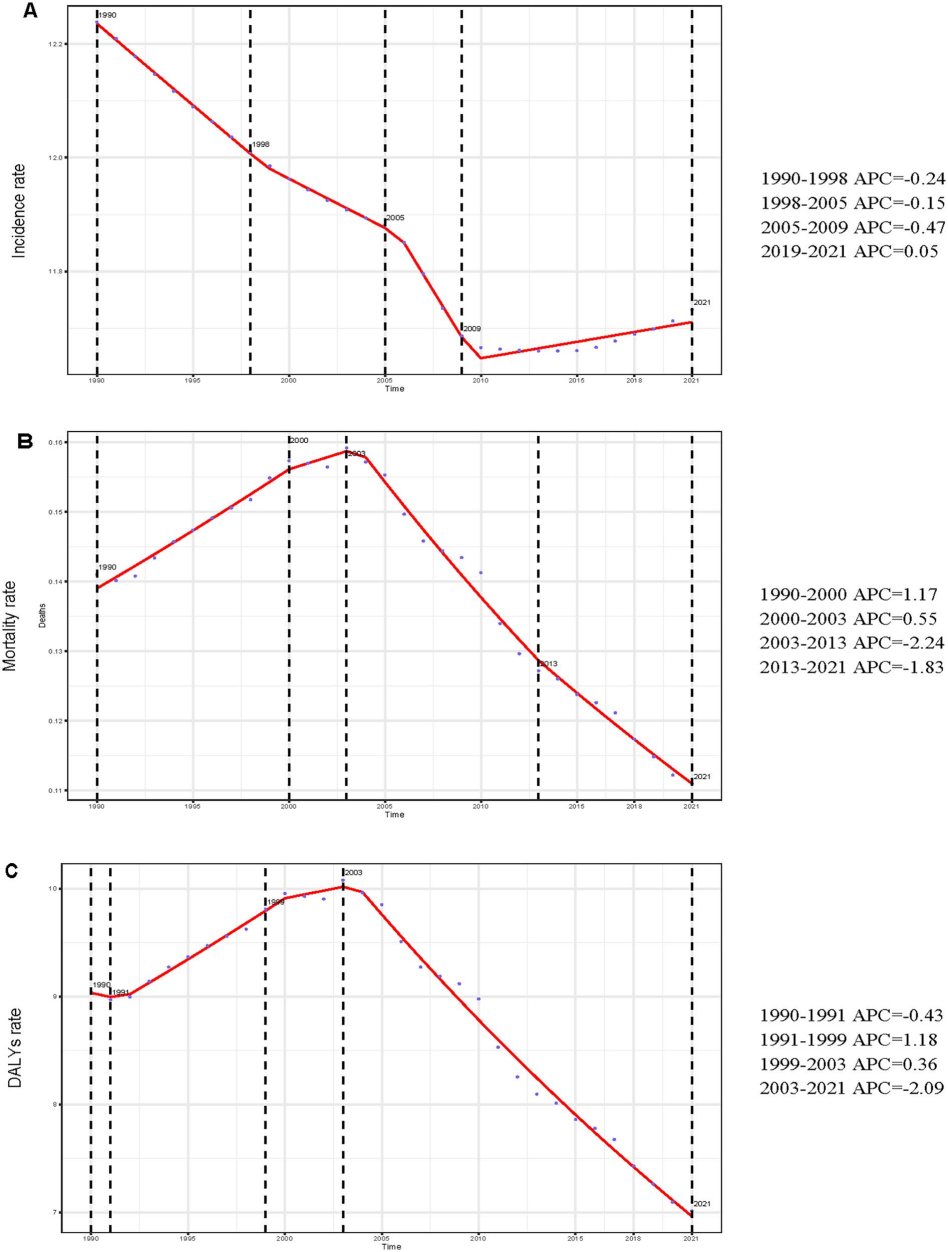
Annual percent change (APC) and trends in global incidence, mortality, and disability-adjusted life years (DALYs) from 1990 to 2021. **(A)** Incidence rate. **(B)** Mortality rate. **(C)** DALYs rate.

**Figure 2:**
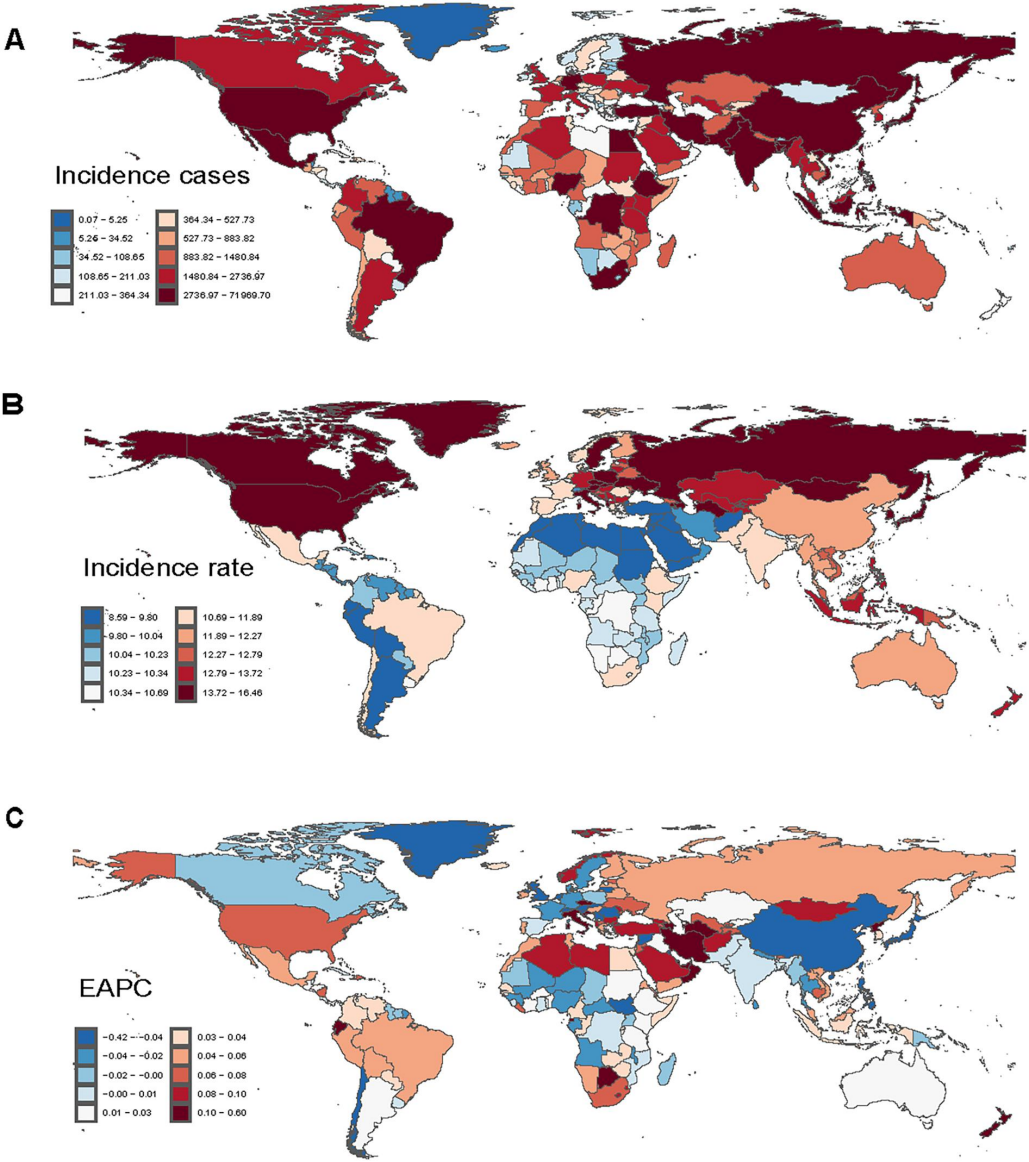
Incidence of myocarditis among adolescents and young adults in 204 countries and territories. **(A)** Number of incidence cases. **(B)** Incidence rate. **(C)** Estimated annual percentage change (EAPC) in incidence.

**Figure 3:**
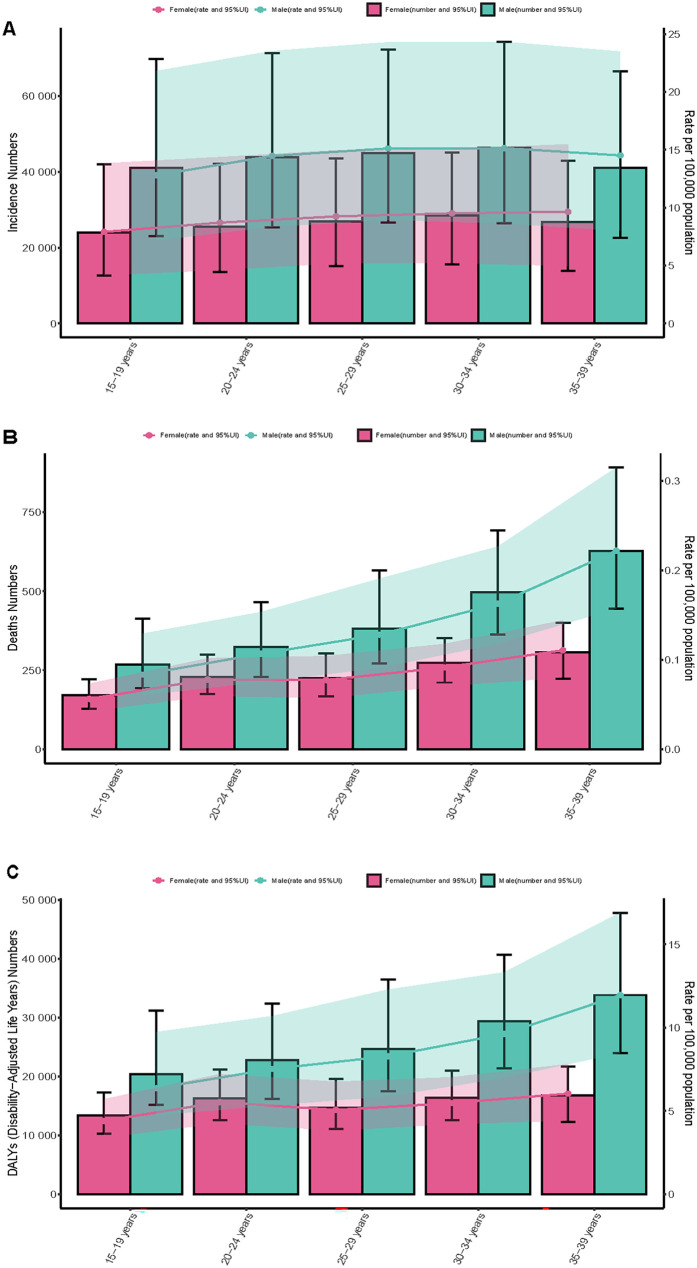
Trends in incidence, mortality, and disability-adjusted life years (DALYs) of myocarditis among adolescents and young adults by age and sex, 1990–2021. **(A)** Incidence case and rate, **(B)** Mortality case and rate, **(C)** DAL Ys case and rate.

**Figure 4:**
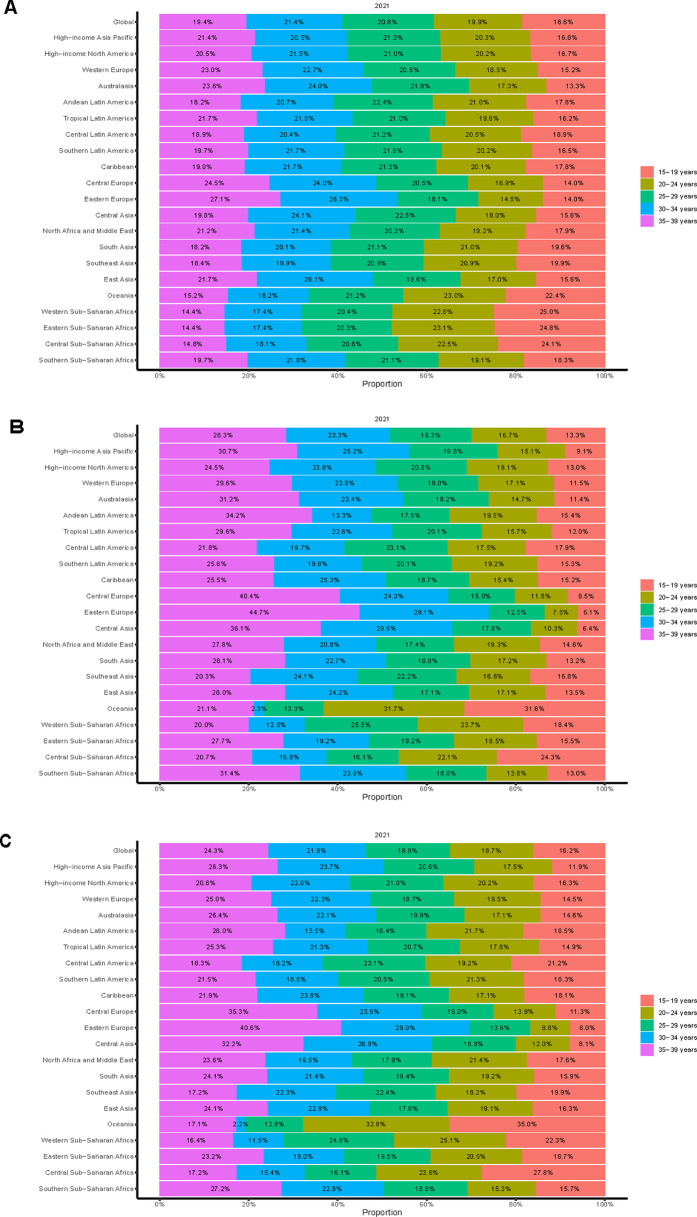
Age-specific percentages of myocarditis incidence, mortality, and disability-adjusted life years (DALYs) among adolescents and young adults in 2021. **(A)** Incidence. **(B)** Deaths. **(C)** DALYs.

Extensive epidemiological data indicate significant temporal variations in the reported incidence trend of myocarditis among adolescents and young adults worldwide. Throughout the study, the estimated incidence rate initially declined before rising slightly, although the overall trend remained downward (APC = −0.14%; 95% CI, −0.17% to −0.12%) (Figure 1A). Notably, a slight increase in the reported incidence rate was observed between 2010 and 2021. Globally, the number of incident myocarditis cases increased by 30.12% (95% UI, 24.62%–35.61%) between 1990 and 2021, rising from 268,236.03 (95% UI, 176,943.09–380,229.53) to 349,033.33 (95% UI, 235,755.91–494,002.44). In contrast, the incidence rate per 100,000 population exhibited a modest decline, decreasing from 12.24 (95% UI, 8.07–17.35) in 1990 to 11.73 (95% UI, 7.93–16.61) in 2021, reflecting an overall reduction of 4.13% (95% UI, −8.18% to −0.08%). According to Table 1, Figure 2A, the EAPC was determined to be −0.16% (95% CI, −0.18% to −0.14%). In 2021, males consistently displayed a higher incidence rate than females across all age subgroups among individuals aged 15–39 years, with the most significant gender disparity observed in the 20–24 age group (Figure 3A). The 30–34-year-old age group noted the highest number of incident cases that year, comprising 21.4% of the total cases within the 15–39 age range. Conversely, the lowest incident cases were noted in the age range of 15–19, which accounted for only 18.6% of the total cases in this population (Figure 4A).

**Table 1:** Incidence of myocarditis in adolescents and young people between 1990 and 2021 at the global and regional level.

Location	Rate per 100,00 (95% UI)						
	1990		2021		1990–2021		
	Incident cases	Incidence rate	Incident cases	Incidence rate	Cases change^b^	Rate change^b^	EAPC^a^
Global	2,68,236.03 (1,76,943.09, 3,80,229.53)	12.24 (8.07, 17.35)	3,49,033.33 (2,35,755.91, 4,94,002.44)	11.73 (7.93, 16.61)	30.12 (24.62, 35.61)	−4.13(−8.18, −0.08)	−0.16(−0.18, −0.14)
SDI							
Low SDI	19,894.51 (12,808.15, 28,456.77)	10.79 (6.95, 15.44)	48,262.19 (31,054.31, 69,024.46)	10.75 (6.92, 15.37)	142.59 (142.10, 143.06)	−0.43(−0.63, −0.24)	−0.01(−0.01, −0.01)
Low-middle SDI	50,794.94 (33,144.97, 72,154.87)	11.20 (7.31, 15.91)	90,112.72 (59,183.31, 1,28,110.98)	11.23 (7.37, 15.96)	77.40 (71.82, 82.53)	0.23(−2.92, 3.13)	0.01 (0.00, 0.01)
Middle SDI	92,269.80 (59,765.99, 1,30,518.49)	12.26 (7.94, 17.34)	1,08,894.92 (73,395.34, 1,54,562.13)	11.74 (7.91, 16.66)	18.02 (9.12, 26.70)	−4.23(−11.45, 2.81)	−0.18(−0.20, −0.15)
High-middle SDI	57,218.60 (37,986.69, 81,029.08)	12.64 (8.39, 17.91)	53,243.10 (36,470.97, 74,432.24)	12.09 (8.28, 16.91)	−6.95(−14.24, −0.11)	−4.35(−11.85, 2.67)	−0.17(−0.19, −0.15)
High SDI	47,818.18 (32,155.47, 67,790.34)	13.78 (9.27, 19.54)	48,250.40 (34,741.85, 65,767.30)	13.66 (9.84, 18.62)	0.90(−4.28, 9.66)	−0.89(−5.98, 7.71)	−0.12(−0.20, −0.05)
Regions							
Andean Latin America	1,475.92 (945.32, 2, 121.56)	9.54 (6.11,13.72)	2,641.34 (1,737.14, 3,806.04)	9.75 (6.41, 14.06)	78.96 (68.97, 87.29)	2.20(−3.51, 6.95)	0.07 (0.07, 0.08)
Australasia	986.19 (668.37, 1,408.21)	12.09 (8.20, 17.27)	1,296.10 (887.76, 1,841.16)	12.38 (8.48, 17.58)	31.42 (26.45, 37.13)	2.34(−1.53, 6.79)	0.12 (0.07, 0.16)
Caribbean	1,469.52 (955.45, 2,129.93)	9.89 (6.43, 14.33)	1,825.48 (1,199.57, 2,618.63)	10.03 (6.59, 14.39)	24.22 (17.97, 29.70)	1.44(−3.66, 5.92)	0.03 (0.02, 0.03)
Central Asia	3,828.06 (2,555.98, 5,387.18)	13.45 (8.98,18.93)	5,135.94 (3,480.41,7,259.51)	13.74 (9.31,19.42)	34.17 (27.56,40.40)	2.11(−2.92,6.85)	0.07 (0.05,0.10)
Central Europe	6,560.52 (4,485.23, 9,174.76)	14.00 (9.57, 19.58)	5,032.93 (3,400.17, 7,097.36)	14.37 (9.71, 20.27)	−23.28(−26.50, −20.59)	2.63(−1.67, 6.23)	0.07 (0.04, 0.10)
Central Latin America	7,124.92 (4,609.42, 10,207.42)	10.44 (6.75, 14.95)	10,684.69 (7,048.28, 15,199.59)	10.56 (6.97, 15.02)	49.96 (41.93, 56.90)	1.20(−4.22, 5.88)	0.03 (0.03, 0.03)
Central Sub-Saharan Africa	2,143.49 (1,378.48, 3,089.16)	10.32 (6.64, 14.88)	5,590.86 (3,607.47, 8,045.43)	10.34 (6.67, 14.87)	160.83 (159.60, 162.41)	0.11(−0.36, 0.72)	0.00 (0.00, 0.00)
East Asia	73,888.59 (47,079.08, 1,04,376.33)	13.06 (8.32, 18.45)	57,642.79 (40,045.87, 80,888.82)	12.03 (8.36, 16.89)	−21.99(−31.06, −12.36)	−7.87(−18.58, 3.49)	−0.35(−0.39, −0.30)
Eastern Europe	11,791.13 (8,048.53, 16,964.23)	13.75 (9.38, 19.78)	9,169.76 (6,222.97, 12,974.39)	13.86 (9.40, 19.61)	−22.23(−25.95, −18.69)	0.80(−4.02, 5.38)	0.06 (0.03, 0.08)
Eastern Sub-Saharan Africa	7,576.93 (4,864.57, 10,912.30)	10.69 (6.86, 15.39)	18,803.58 (12,082.30, 27,035.59)	10.73 (6.90, 15.43)	148.17 (146.86, 149.57)	0.42(−0.11, 0.99)	0.02 (0.01, 0.02)
High-income Asia Pacific	10,536.36 (7,101.93, 14,721.11)	15.61 (10.52, 21.81)	7,613.96 (5,353.10, 10,492.47)	15.07 (10.59, 20.76)	−27.74(−31.21, −23.59)	−3.49(−8.14, 2.04)	−0.21(−0.27, −0.16)
High-income North America	16,060.70 (10,587.90, 22,916.46)	14.17 (9.34, 20.22)	18,550.61 (13,848.45, 24,633.88)	15.06 (11.24, 20.00)	15.50 (2.63, 35.66)	6.25(−5.59, 24.79)	0.07(−0.12, 0.27)
North Africa and Middle East	12,378.06 (8,042.24, 17,545.86)	9.25 (6.01, 13.11)	23,869.18 (15,925.97, 33,482.86)	9.39 (6.26, 13.17)	92.83 (79.45, 105.88)	1.50(−5.55, 8.36)	0.07 (0.06, 0.08)
Oceania	327.97 (216.05, 467.23)	12.35 (8.13, 17.59)	695.19 (459.65, 986.84)	12.34 (8.16, 17.51)	111.97 (106.73, 116.82)	−0.07(−2.54, 2.22)	−0.00(−0.00, 0.00)
South Asia	49,523.10 (32,175.35, 70,641.28)	11.47 (7.45,16.37)	91,301.01 (59,819.98, 1,29,678.31)	11.54 (7.56, 16.40)	84.36 (79.21, 89.08)	0.61(−2.21, 3.18)	0.02 (0.01, 0.02)
Southeast Asia	25,412.53 (16,736.83, 36,010.65)	12.90 (8.50,18.28)	35,979.46 (24,150.96,50,770.83)	12.97 (8.71,18.31)	41.58 (34.76,48.34)	0.58(−4.27,5.38)	0.01 (0.00,0.02)
Southern Latin America	1,954.19 (1,318.65, 2,763.49)	10.24 (6.91,14.48)	2,641.10 (1,855.17, 3,645.68)	10.24 (7.19, 14.13)	35.15 (28.73, 44.36)	−0.04(−4.79, 6.77)	−0.03(−0.05, −0.01)
Southern Sub-Saharan Africa	2,409.42 (1,564.26, 3,436.14)	11.15 (7.24, 15.90)	3,858.50 (2,540.39, 5,480.18)	11.34 (7.46, 16.10)	60.14 (50.16, 69.62)	1.70(−4.64, 7.72)	0.07 (0.06, 0.08)
Tropical Latin America	7,098.29 (4,669.13, 10,135.41)	11.04 (7.26,15.76)	9,875.46 (6,608.34, 14,111.24)	11.18 (7.48, 15.98)	39.12 (30.77, 46.46)	1.32(−4.76, 6.66)	0.05 (0.04, 0.05)
Western Europe	17,935.26 (12,198.72, 25,445.44)	12.44 (8.46, 17.66)	16,195.98 (11,192.57, 22,595.13)	12.48 (8.62, 17.41)	−9.70(−12.11, −7.09)	0.29(−2.39, 3.18)	−0.01(−0.04, 0.02)
Western Sub-Saharan Africa	7,754.88 (4,993.25,11,108.36)	10.83(6.98, 15.52)	20,629.40(13,251.67, 29,583.16)	10.79(6.93, 15.47)	166.02(164.51, 167.72)	−0.42(−0.99, 0.21)	−0.01(−0.02, −0.01)

EAPC, estimated annual percentage change; SDI, sociodemographic Index; UI, uncertainty interval. An EAPC is expressed as:

^a^ 95% confidence interval.

^b^ Change shows the percentage change.

#### Mortality

Over the past three decades, the estimated global mortality rate of myocarditis among adolescents and young adults has shown a fluctuating trend, initially increasing and subsequently declining (Figure 1B). Aligning with the general trend observed in incidence, the mortality rate for this age group demonstrated an overall decrease, with an APC of −0.71% (95% CI, −1.08% to −0.34%). Myocarditis-related deaths in adolescents and young adults increased by 6.80% (95% UI, −18.72% to 30.57%) between 1990 and 2021, from 3,090.62 cases (95% UI, 2,402.23–3,917.61) to 3,300.73 cases (95% UI, 2,643.01–4,256.11). The mortality rate, on the other hand, decreased over the same period, falling from 0.14 per 100,000 people (95% UI, 0.11–0.18) in 1990 to 0.11 per 100,000 (95% UI, 0.09–0.14) in 2021, a 21.31% decrease (95% UI, −40.11% to −3.80%). According to Supplementary Table S1, Figure 2B, the EAPC was −0.88% (95% CI, −1.16% to −0.59%). Males had a continuously higher death rate from myocarditis than females in 2021 throughout all age groups between 15 and 39 years, with the gender gap being particularly noticeable in the 35–39 age range (Figure 3B). This age group also recorded the highest number of myocarditis-related deaths that year, contributing to 28.3% of total mortality cases within the 15–39 age range. In contrast, individuals aged 15–19 exhibited the lowest number of deaths due to myocarditis, representing only 13.3% of the total myocarditis-related deaths among adolescents and young adults aged 15–39 years (Figure 4B).

#### DALYs

Notably throughout the last 30 years, there has been a rising and then falling trend in the estimated worldwide DALYs rate for myocarditis in adolescents and young adults (Figure 1C). The global DALYs rate among adolescents and young adults has also exhibited an overall declining trend (APC = −0.82%; 95% CI, −1.19% to −0.46%), consistent with the general trends in incidence and mortality. There was a 5.27% (95% UI, −19.37% to 27.55%) rise in the number of myocarditis DALYs among adolescents and young adults worldwide between 1990 and 2021, from 198,205.70 (95% UI, 155,684.08–24,9629.06) in 1990–208,644.28 (95% UI, 169,653.92–26,518.34) in 2021. In addition, the DALYs rate decreased from 9.04 (95% UI, 7.10–11.39) per 100,000 in 1990 to 7.01 (95% UI, 5.70–8.99) per 100,000 in 2021, an overall decrease of 22.44% (95% UI, −40.59% to −6.02%). According to Supplementary Table S2, Figure 2C, the EAPC was −0.90% (95% CI, −1.17% to −0.62%). In 2021, males exhibited higher rates of myocarditis-related DALYs than females across all age groups from 15 to 39 years, with the most pronounced sex disparity observed in individuals aged 35–39 years (Figure 3C). Notably, the 35–39 age group had the highest DALYs cases of myocarditis in 2021, accounting for 24. 3% of all DALYs cases in 15–39 years in 2021. On the other hand, myocarditis patients in the 15–19 age range had the fewest DALYs, making up just 16.2% of all DALYs cases in the same year among those aged 15–39 (Figure 4C).

#### SDI region level

The middle SDI region in 2021 had the greatest number of cases of incidence (108,894.92; 95% UI, 73,395.34–154,562.13), deaths (1,135.47; 95% UI, 807.86–1,385.40), and DALYs (71,567.92; 95% UI, 51,412.21–87,197.95) among the five SDI regions. Between 1990 and 2021, low SDI regions experienced the most substantial increases, with a 142.59% rise in incident cases (95% UI, 142.10%–143.06%), an 82.75% increase in mortality (95% UI, 32.66%–163.06%), and an 84.62% rise in DALYs (95% UI, 142.10%–143.06%). The greatest mortality rate (0.15 per 100,000; 95% UI, 0.12–0.18) and DALYs rate (9.46 per 100,000; 95% UI, 7.42–11.00) were found in high-middle SDI locations, whereas the highest incidence rate (13.66 per 100,000; 95% UI, 9.84–18.62) was found in high SDI regions. The middle SDI region demonstrated the largest annual declines across all indicators, recording the lowest EAPCs in incidence (−0.18%; 95% CI, −0.20 to −0.15), mortality (−1.09%; 95% CI, −1.38 to −0.79), and DALYs (−1.13%; 95% CI, −1.40 to −0.85) (Table 1; Supplementary Tables S1, S2; Supplementary Figure S1).

### Geographic regional trends

#### Incidence

**Figure 5:**
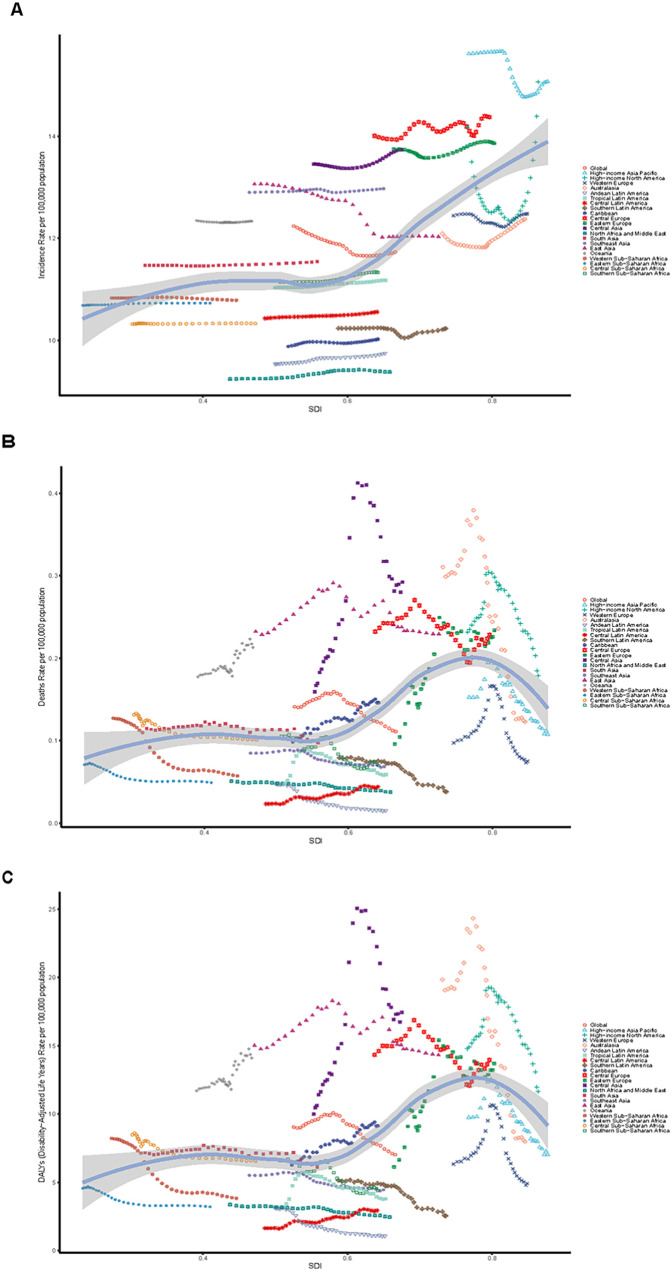
Association between incidence, mortality, and disability-adjusted life years (DALYs) rates of myocarditis and regional Sociodemographic Index (SDI), 1990–2021. **(A)** Incidence rate. **(B)** Mortality rate. **(C)** DALYs rate.

With 91,301.01 (95% UI, 59,819.98–129,678.31) incident cases, South Asia had the most of the 21 worldwide regions in 2021, while Oceania had the fewest, with just 695.19 cases (95% UI, 459.65–986.84). North Africa and the Middle East had the lowest incidence rate, at 9.39 per 10,000 people (95% UI, 6.26–13.17), whereas the High-income Asia Pacific region reported the highest rate, at 15.07 per 10,000 population (95% UI, 10.59–20.76). Between 1990 and 2021, Australasia experienced the greatest increase in incidence rate, with an EAPC of 0.12% (95% CI, 0.07–0.16), whereas East Asia exhibited the largest decline (EAPC = −0.35%; 95% CI, −0.39 to −0.30). In 2021, the global incidence rate was estimated at 11.73 per 10,000 population (95% UI, 7.93–16.61). The incidence rates were lower than the global mean in twelve areas, including Andean Latin America, the Middle East, and North Africa, while they were higher in nine areas, including High-income Asia Pacific and High-income North America (Table 1; Figure 5A).

#### Mortality

With 1,097.59 (95% UI, 726.99–1,426.58) myocarditis-related deaths in 2021, East Asia had the largest number, while Andean Latin America had the lowest, with just 4.05 deaths (95% UI, 2.78–5.78). In terms of mortality rates, Central Asia recorded the greatest value at 0.29 per 10,000 population (95% UI, 0.22–0.37), while Andean Latin America reported the lowest at 0.01 per 10,000 people (95% UI, 0.01–0.02). Eastern Europe saw the largest increase in mortality rate between 1990 and 2021, with an EAPC of 2.32% (95% CI, 1.76–2.89), while Andean Latin America experienced the most significant decline (EAPC = −4.31%; 95% CI, −4.62 to −3.99). Globally, the mortality rate for myocarditis in 2021 was 0.11 per 10,000 population (95% UI, 0.09–0.14). Thirteen regions, including Andean Latin America, reported mortality rates below the global average, while eight regions, such as Central Asia and Central Europe, had rates above the global average (Supplementary Table S1; Figure 5B).

#### DALYs

The number of DALYs-related myocarditis was highest in East Asia (68,601.16; 95% UI, 46,213.93–88,361.53), and lowest in Andean Latin America (287.09; 95% UI, 204.73–390.49), which is similar to the mortality in 2021. Andean Latin America had the lowest DALY rates at 1.06 per 10,000 people (95% UI, 0.76–1.44), whereas Central Asia reported the largest at 17.43 per 10,000 people (95% UI, 13.33–22.19). Central Latin America experienced the largest increase in DALYs rate, with an EAPC of 2.09% (95% CI, 1.89–2.30), while Andean Latin America saw the greatest decrease (EAPC = −4.01%; 95% CI, −4.31 to −3.71) between 1990 and 2021. Globally, the DALYs rate for myocarditis in 2021 was 7.01 per 10,000 population (95% UI, 5.70–8.99). Thirteen regions, including Andean Latin America and North Africa & the Middle East, reported DALYs rates below the global average, while eight regions, such as Central Asia and East Asia, had rates exceeding the global average (Supplementary Table S2; Figure 5C).

### National trends

#### Incidence

With 71,257.12 (95% UI, 46,817.01–100,807.05) incident cases, India recorded the most in 2021, while Tokelau had the fewest, only having 0.06 cases (95% UI, 0.04–0.09). Regarding incidence rates per 10,000 population, Poland ranked highest at 16.30 (95% UI, 10.97–23.09), while the Syrian Arab Republic reported the lowest rate, recorded at 8.68 per 100,000 population (95% UI, 5.46–12.53). Furthermore, with an EAPC of 10.47% (95% CI, 8.40–12.58) between 1990 and 2021, New Zealand showed the largest rise in incidence rate (Supplementary Table S3; Figure 2).

#### Mortality

Myocarditis-related deaths in 2021 were highest in the Central African Republic (1,058.50 cases, 95% UI, 691.43–1,389.86), while Guyana had the highest mortality rate (0.90 per 10,000 people, 95% UI, 0.65–1.18). Additionally, between 1990 and 2021, Italy had the largest decrease in mortality rate (EAPC = −7.79%; 95% CI, −9.06 to −6.50) and Kazakhstan had the most rise (EAPC = 10.78%; 95% CI, 8.68–12.92) (Supplementary Table S4; Supplementary Figure S2).

#### DALYs

In 2021, China recorded the highest number of DALYs globally, with 66,143.95 cases (95% UI, 43,846.28–85,997.39). With just 0.02 DALY cases (95% UI, 0.01–0.02), the Cook Islands, on the other hand, had the fewest. Guyana reported the highest DALYs rate worldwide at 54.78 per 10,000 population (95% UI, 39.76–71.90), whereas Egypt recorded the smallest, at 0.26 per 10,000 people (95% UI, 0.13–0.52). Kazakhstan's DALYs rate increased the highest between 1990 and 2021 (EAPC = 10.47%; 95% CI, 8.40–12.58), while Italy's rate decreased the most (EAPC = −7.39%; 95% CI, −8.57 to −6.20) (Supplementary Table S5; Supplementary Figure S3).

### Frontier analysis

**Figure 6:**
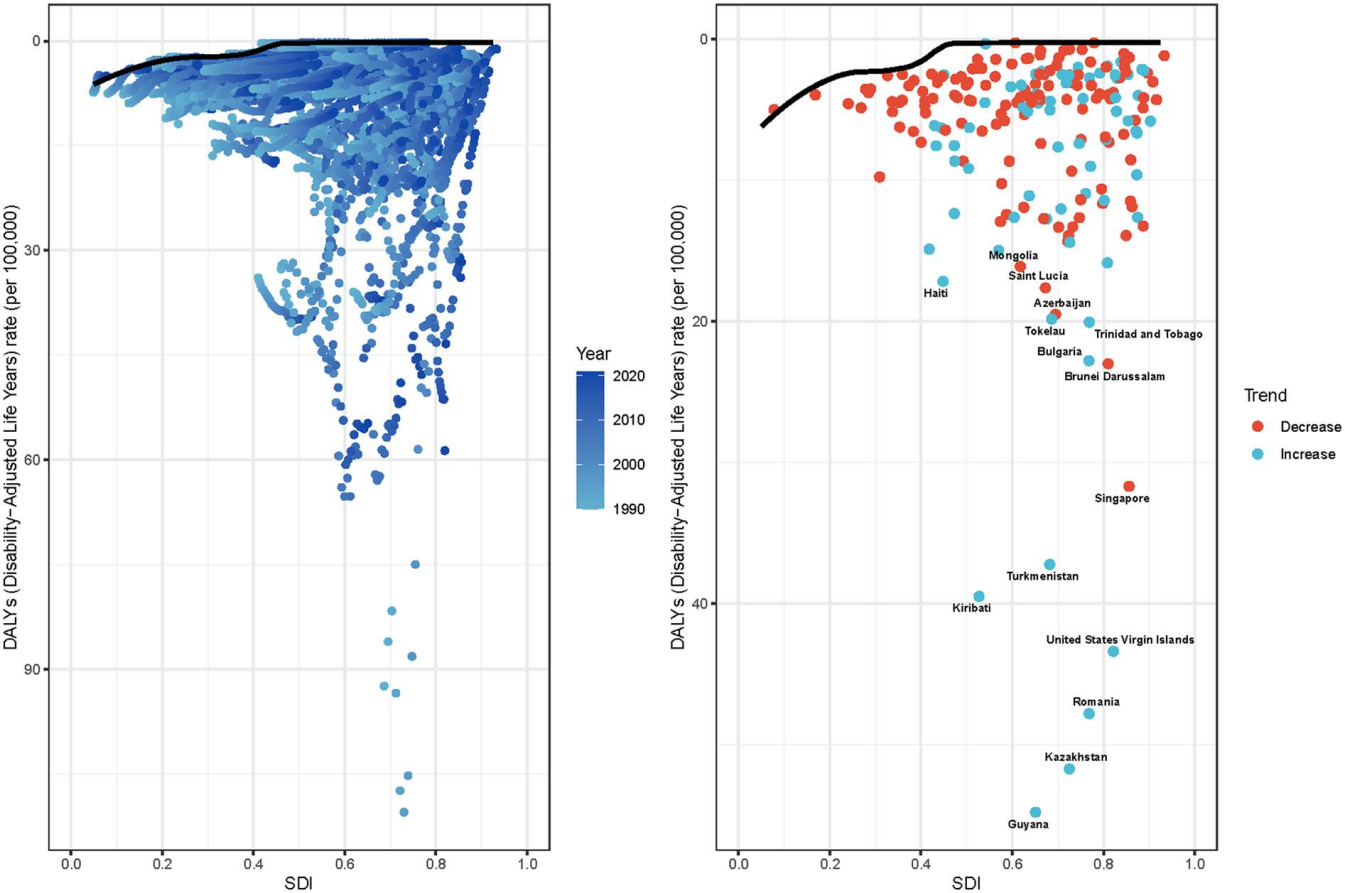
Frontier analysis exploring the relationship between SDI and DALYs for myocarditis among adolescents and young adults in 204 countries and territories. In Figures a, the color change from light blue (1990) to dark blue (2021) represents the change in years. In Figures b, each point represents a specific country or territory in 2021, the frontier line is shown in black, and the top 15 countries and territories with the largest differences from the frontier are marked in black. The direction of DALYs rate change from 1990 to 2021 is indicated by the color of the dots, with red dots representing decreases and blue dots representing increases. SDI, sociodemographic index.

We performed a frontier analysis using the DALYs rate and the SDI on data from 1990 to 2021 to determine where there may be room for improvement in lowering the burden of myocarditis in adolescents and young adults while accounting for regional and national development levels (Supplementary Table S6 and Figure 6). 15 nations and areas with the biggest actual disparities in potential improvement (effective difference range, 58.53–15.85) were found by the analysis. These included Guyana, Kazakhstan, Romania, the United States Virgin Islands, Kiribati, Turkmenistan, Singapore, Brunei Darussalam, Bulgaria, Trinidad and Tobago, Tokelau, Azerbaijan, Saint Lucia, Haiti, and Mongolia. Somalia, Niger, Chad, Mali, South Sudan, and Burkina Faso were among the frontier nations and areas with low SDI. On the other hand, Sweden, New Zealand, and the Russian Federation had high SDI levels but were still demonstrating significant potential for improvement concerning their level of development. There may be space for improvement in lowering the burden of myocarditis among adolescents and young adults across different nations and areas, according to frontier analysis. Despite severe resource constraints, several low-SDI nations, like Somalia and Niger, have demonstrated significant effectiveness in controlling the disease burden.

### Risk factors

**Figure 7:**
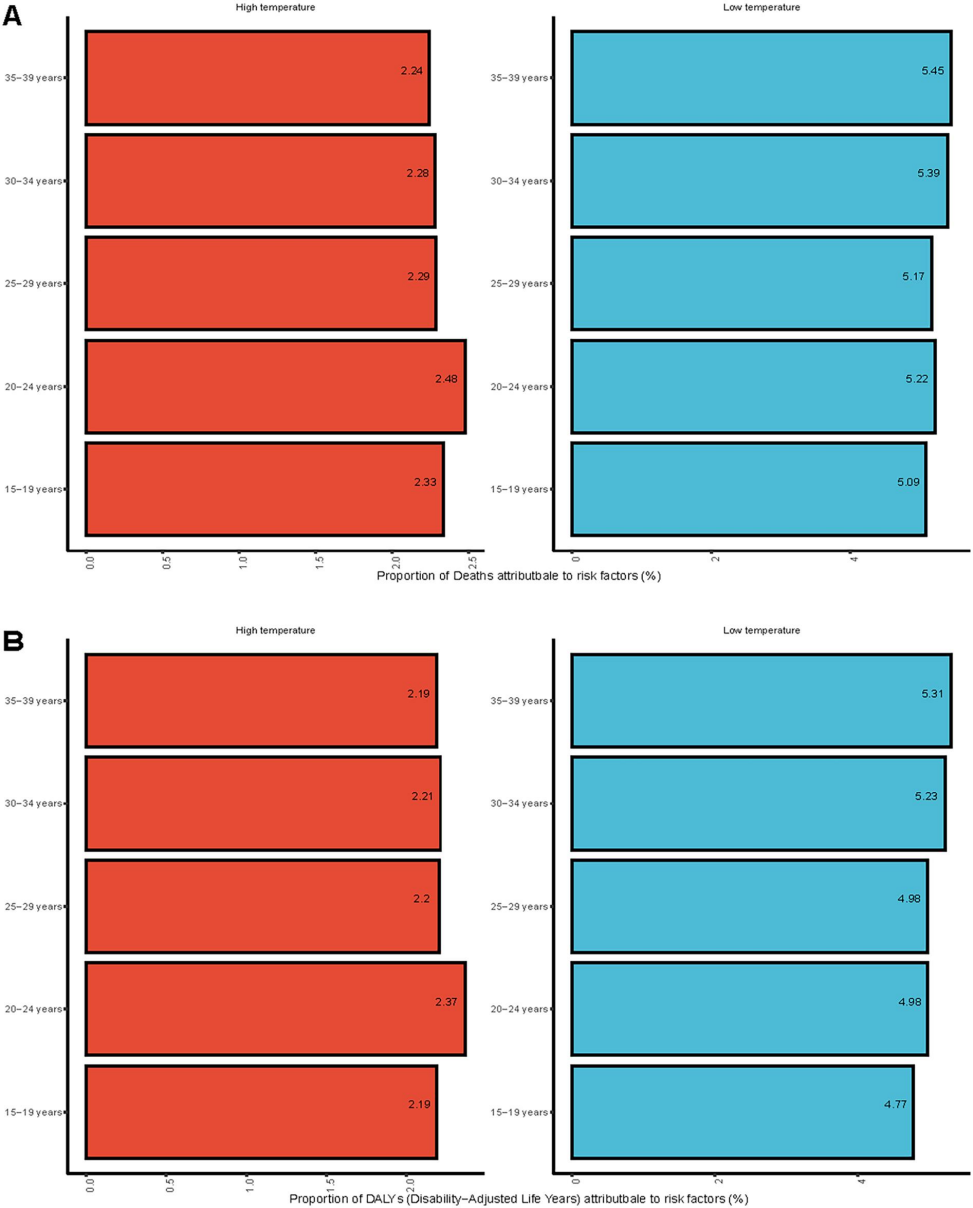
Proportion of myocarditis among mortality and DALYs risk factors for adolescents and young adults in 2021. **(A)** Mortality. **(B)** DALYs.

Based on the 2021 GBD database, there are two main environmental risk factors identified at the population level associated with myocarditis in adolescents and young adults: high temperature and low temperature. In 2021, individuals aged 20–24 represented the largest proportion of deaths and DALYs related to high temperatures. Conversely, the greatest burden from low temperatures was seen in the 35–39 age group (Figure 7). Between 1990 and 2021, global and regional trends in temperature-related mortality and DALYs underwent substantial shifts. Globally, 2.31% of myocarditis-related mortality was attributed to high temperature, while 5.30% was due to low temperature. Similarly, high and low temperatures contributed to 2.23% and 5.08% of DALYs, respectively. Across all five SDI regions, both the proportion of mortality and the DALYs attributed to high temperature exceeded those associated with low temperature, suggesting a greater overall health burden from high temperature (Supplementary Figure S4). In 2021, the regions with the largest percentages of deaths attributable to high temperatures were North Africa, and Middle East, and South Asia. Conversely, the largest percentages of deaths linked to low temperatures were found in Central Europe and Western Europe. Similarly, the regions of North Africa, and Middle East, and South Asia had the highest percentages of DALYs in 2021 that were attributed to high temperatures. In contrast, Central Europe and Western Europe ranked among the top regions for DALYs associated with low temperatures (Supplementary Figure S5).

### Predictions for myocarditis in adolescents and young adults

**Figure 8:**
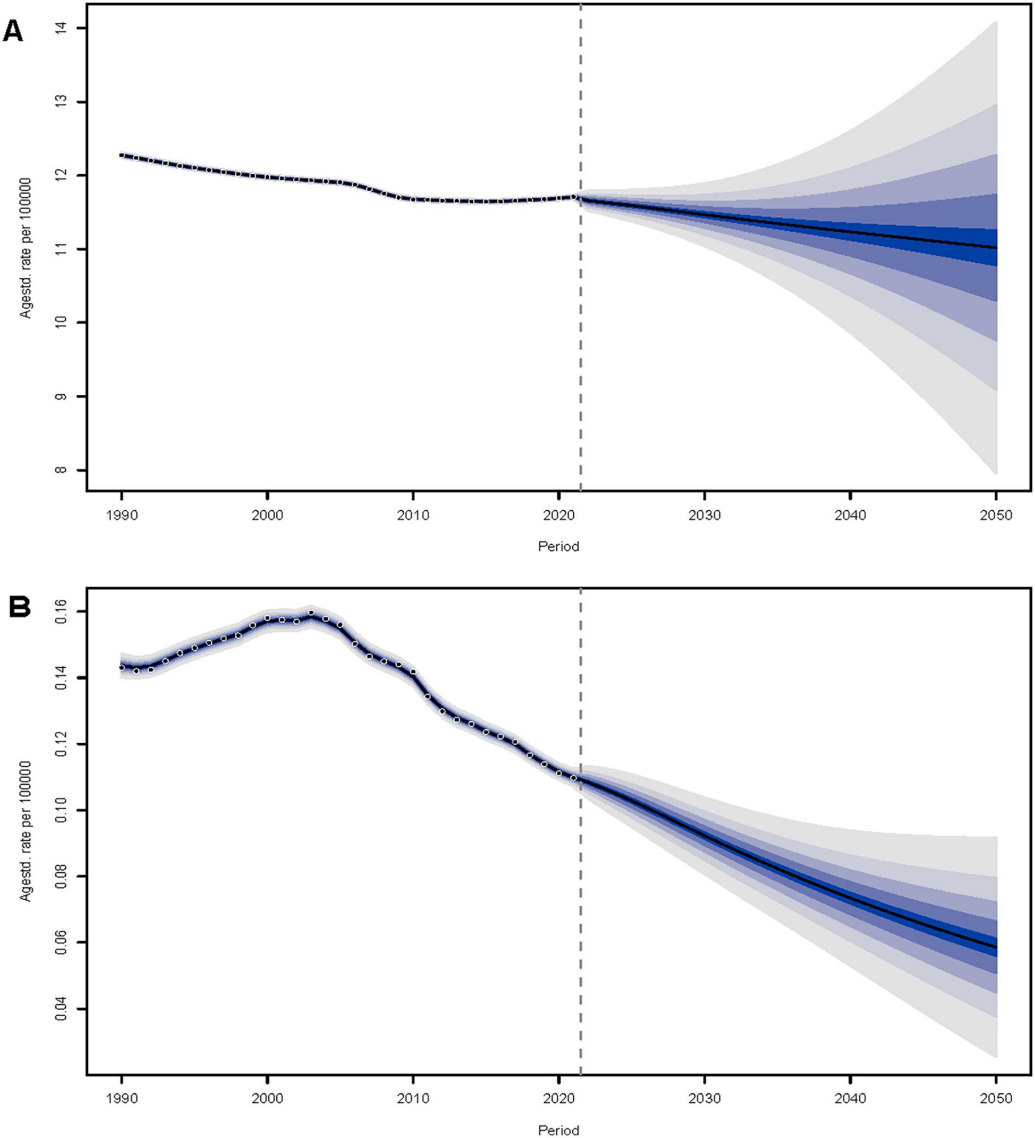
The prediction of myocarditis for adolescents and young adults in incidence and mortality rate from 2022 to 2050. **(A)** Incidence. **(B)** Mortality.

The BAPC model was employed to forecast future trends in myocarditis incidence and mortality rates among people aged 15–39 between 2022 and 2050. The results indicate a continuous decline in both rates over the projected period (Figure 8). Furthermore, predictions based on the BAPC model were made for particular age groups (15–19, 20–24, 25–29, 30–34, and 35–39 years), and during the forecast period, a consistent downward trend in the incidence and mortality rates of myocarditis was noted for all groups (Supplementary Figure S6). The 35–39 age group is expected to continue to have a relatively higher incidence rate than the younger age groups for the duration of the projection period, despite the overall drop in incidence. Regarding mortality, although a reduction is anticipated over time, the 35–39 age group exhibited the highest mortality rate, and it is expected to remain elevated relative to other age groups.

## Discussion

Adolescents and young adults’ physical and mental health have been severely impacted by myocarditis during the last three decades. With rising healthcare expenditures and broader societal implications, it has emerged as a critical public health concern. It is essential to get a thorough understanding of the incidence, mortality, and DALYs of myocarditis in this group in order to create effective public health interventions. However, to date, no longitudinal epidemiological analysis specifically addressing myocarditis among adolescents and young adults, either at the global, regional, or national level, has been conducted. This study is the first to analyze global trends in incidence, mortality, DALYs, and related risk factors among this demographic using GBD data spanning 1990 to 2021. The results are intended to offer solid evidence to assist policymakers and healthcare providers in formulating effective strategies for prevention and management.

The results indicate that from 1990 to 2021, the global incidence, mortality, and DALYs rates for myocarditis demonstrated a consistent decline, with EAPCs of −0.16%, −0.88%, and −0.90%, respectively. These trends align with previous global assessments, suggesting a reduced health burden among adolescents and young adults (4). Several key factors have likely contributed to this decline. It should be noted that these declining trends reflect GBD-estimated and reported rates, and may partly be influenced by temporal changes in diagnostic practices and disease surveillance capacity. In addition, evolving diagnostic criteria and ICD coding practices for myocarditis across countries and over time may affect comparability, particularly in low-resource settings where advanced diagnostic tools are less available. Chief among them is the advancement in diagnostic capabilities, particularly with the expanded use of cardiac magnetic resonance imaging (CMR), high-sensitivity troponin assays, and ambulatory electrocardiographic monitoring (19, 20). These technologies enable earlier detection, allowing for timely medical interventions that reduce both mortality and long-term disability. Furthermore, enhanced treatment strategies, such as more precise glucocorticosteroid and immunosuppressant regimens, coupled with improved management of complications like chronic heart failure, have contributed to better disease outcomes (21, 22). Public health efforts have also been instrumental, with increased cardiovascular health education fostering greater awareness of key symptoms and encouraging earlier healthcare consultation, which has led to more standardized treatment practices.

However, from 2010 to 2021, there was a notable increase in myocarditis incidence within this demographic. This rise may be partly attributed to the resurgence of certain viral infections, such as influenza, Epstein–Barr virus (EBV), and enteroviruses. Additionally, the increase in myocarditis cases following COVID-19 infection or vaccination, particularly among adolescent males, could also explain this upward trend (23). Despite the overall decline in age-standardized rates, the absolute number of incident cases, deaths, and DALYs increased by 30.12%, 6.80%, and 5.27%, respectively, during the same period. This apparent discrepancy likely reflects improved detection, particularly of milder or subclinical cases, due to broader access to advanced diagnostic tools like CMR and high-sensitivity cardiac biomarkers (22). Therefore, the observed increase in incidence during this period should be interpreted with caution, as it may not solely represent a true rise in disease risk but also changes in detection intensity and reporting practices. Moreover, inadequate long-term management of sequelae, such as dilated cardiomyopathy, may have contributed to persistent functional impairment, thereby sustaining the DALY burden (24). In conclusion, while the age-standardized rates for myocarditis have declined among adolescents and young adults globally, the growing absolute case burden and the ongoing impact of sequelae underscore the need for enhanced early screening, more effective interventions, and robust long-term care strategies.

Our study notably revealed significant gender-related differences in the incidence of myocarditis. Specifically, among individuals aged 15–39 years, males consistently exhibited higher rates of myocarditis incidence, mortality, and DALYs compared to females. These findings are consistent with previous studies. One such study reported a male-to-female prevalence ratio for myocarditis ranging from 1.5:1 to 1.7:1 (25). Another study observed that 77% of the 3,198 myocarditis patients were male (26). These differences may be partially explained by sex hormone levels and immune response variations. For instance, viral replication in the myocardium tends to be higher in males, potentially due to testosterone's role in amplifying myocardial inflammation by upregulating pro-inflammatory cytokines such as IL-1β and TNF-α (27). Conversely, estrogen appears to exert a cardioprotective effect by promoting anti-inflammatory pathways and enhancing cardiomyocyte repair (28). The clinical presentation also differs between genders. Men often experience classic symptoms such as chest pain and markedly elevated troponin levels, facilitating early diagnosis, although they are more likely to progress to severe forms. In contrast, women frequently report non-specific symptoms like fatigue or palpitations, which are prone to misdiagnosis as anxiety or functional cardiac disorders, increasing the risk of delayed or missed diagnosis (29). Therefore, incorporating a gender-sensitive perspective into research, clinical decision-making, and public health strategies is essential to reduce both the underdiagnosis in women and the severity in men.

Age-related variation showed that incident cases, which made up the largest percentage of all cases, peaked in the 30–34 age range. This may be linked to lifestyle stressors, including career development and parenting responsibilities, which are prevalent in this demographic (30). In the meantime, the number of deaths and DALYs peaked in people between the ages of 35 and 39, most likely as a result of growing cardiac damage and an elevated risk of long-term problems or death as people age. On the other hand, because of improved myocardial regenerating capacity and higher baseline cardiovascular health, the 15–19 age group exhibited the lowest proportions of cases of incidence, death, and DALYs. These findings suggest the need for targeted prevention strategies, such as routine screening for myocardial injury markers after intense physical activity in individuals aged 30–34, integrated comorbidity management (e.g., smoking cessation, early detection of heart failure) for those aged 35–39, and improved diagnostic accuracy through non-invasive methods like high-sensitivity troponin testing and cardiac MRI in the 15–19 age group. In individuals aged 30–39 presenting with chest pain or dyspnea, myocarditis should be considered in the differential diagnosis rather than attributing symptoms solely to psychosocial stress or suboptimal health status.

In 2021, significant disparities in the epidemiological characteristics of myocarditis among adolescents and young adults were observed across global regions classified by the SDI. Notably, regions with a high SDI demonstrated the highest incidence rates, a finding consistent with earlier reports (4, 31). This pattern might be explained by the existence of robust screening systems and the extensive use of cutting-edge diagnostic technologies, especially cardiac magnetic resonance imaging, which improves the detection of myocarditis patients (32). On the other hand, the low SDI region saw the most noticeable increases in incidence, mortality, and DALY between 1990 and 2021. These patterns are probably caused by issues like undeveloped healthcare systems, insufficient service coverage, and an absence of successful early detection initiatives. At the same time, regions with a middle SDI reported the highest absolute numbers of incidence, deaths, and DALYs. However, the middle SDI region also demonstrated the lowest EAPC across all SDI groups, indicating a slowdown in the growth of disease burden. This decline could be ascribed to the introduction of public health initiatives and heightened health awareness. Based on these findings, it is crucial to prioritize low and middle SDI regions in future global efforts to prevent and control myocarditis. It is important to emphasize that SDI is a population-level indicator, and the observed associations reflect regional characteristics rather than individual-level risk. Therefore, these findings should not be interpreted as direct causal relationships at the individual level.

Regionally, Australasia showed the greatest annual increase in incidence rate, potentially indicating either a rising risk of disease or improved surveillance and diagnostic accuracy in the area. Because of its vast population, rapid aging, and high burden of cardiovascular comorbidities, East Asia recorded the largest number of myocarditis-related mortality and DALYs. Additionally, the efficient case documentation and identification systems in East Asian countries may also contribute to this pattern. Due to its large population and high burden of infectious illnesses, India had the most incident cases globally at the national level in 2021 (33). Conversely, New Zealand recorded the largest EAPC incidence rate, which may reflect notable improvements in screening practices, strategy implementation, and clinical awareness of myocarditis. In the future, greater emphasis should be placed on the early identification and standardized management of high-risk groups. Moreover, incorporating myocarditis into chronic disease prevention and control frameworks may contribute to reducing its overall disease burden.

This study used frontier analysis to assess data from 204 nations and territories in order to explore the potential for reducing the burden of myocarditis among adolescents and young adults. Findings revealed that the 15 countries or regions with the greatest gaps between achievable outcomes and current disease burden warrant prioritized attention in future public health planning. It's interesting to note that despite having acute shortages of healthcare resources, several low-SDI nations or territories, like Somalia, Niger, and Chad, have made significant progress in lessening the impact of myocarditis. These outcomes may reflect the implementation of targeted interventions or adaptive health policies in resource-constrained settings. Conversely, some high-SDI nations, including the Russian Federation and Sweden, performed below expectations in managing myocarditis burden among youth and young adults (34). Although these countries possess advanced screening technologies and established early intervention systems, challenges such as suboptimal resource utilization and insufficiently targeted risk-group management may undermine intervention outcomes. Accordingly, refining health system strategies to enhance the efficiency of screening coverage and standardize care for vulnerable groups remains an urgent priority. It is important to acknowledge that disparities still exist across nations in terms of diagnostic capability, public awareness of myocarditis, and access to essential healthcare services. Continued efforts to improve the formulation and execution of national health policies, alongside the promotion of population-specific, data-informed prevention strategies, will be key to effectively reducing the disease burden (35).

The GBD 2021 database identified high and low temperatures as two major risk factors contributing to myocarditis-related mortality and DALYs among adolescents and young adults. The study indicated that high temperature-related deaths and DALYs peaked in individuals aged 20–24, whereas low temperature-related outcomes were greatest among those aged 35–39. These findings highlight potential age-related differences in vulnerability to temperature extremes among young populations affected by myocarditis. A global assessment of temperature-related disease burdens estimated that in 2019, approximately 169 million deaths were attributable to temperature exposure, with North Africa and Middle East, and South Asia carrying the highest burden of heat-related mortality, and Eastern Europe and Central Europe experiencing the highest burden from low temperature (36). These trends are consistent with our findings regarding regional variations in temperature-associated myocarditis burden. Moreover, a systematic review examining the impact of meteorological variables on human mortality concluded that both moderately cold and extremely hot temperatures significantly raise the chance of dying from respiratory and cardiovascular diseases (37). The underlying mechanisms may involve physiological changes triggered by abnormal body temperatures, including elevated blood and plasma viscosity, increased erythrocyte concentrations, and upregulation of specific proteins, which together may lead to enhanced contractility of the cardiovascular system, raising the likelihood of cardiovascular events (38). Overall, the findings underscore the significance of exposure to extreme temperatures as an environmental risk factor that can be modified. In the context of continuous global climate change, it will be critical to incorporate temperature into preventative frameworks for environmentally sensitive illnesses such as myocarditis, especially by establishing climate-responsive and region-specific public health interventions. As these temperature-related risk estimates are derived from population-level exposure data, they may not fully capture individual susceptibility. Further studies incorporating individual-level exposure and clinical data are needed to clarify causal mechanisms.

According to projections based on the BAPC model, between 2022 and 2050, the incidence rate and mortality rate of myocarditis among people aged 15–39 will continue to fall. This decreasing trend is consistently observed across all five age subgroups. However, it is noteworthy that the 35–39 age group is predicted to sustain a relatively elevated incidence rate throughout the forecast period, despite the overall decline. Regarding mortality, while a downward trajectory is also projected for this group, historical data show that individuals aged 35–39 have consistently exhibited the highest mortality rate among all age brackets. These findings imply that individuals in the upper age range of the adolescent and young adult population may face substantially greater health vulnerability, potentially due to cumulative environmental exposures, adverse behavioral patterns, or the presence of chronic comorbidities over time (39). In light of these observations, although the overall disease burden is expected to decrease, there remains a critical need to establish age-tailored prevention and early intervention strategies. Special attention should be directed toward improving risk identification and ensuring adequate resource allocation for the 35–39 age cohort, thereby promoting further reduction in the burden of myocarditis.

### Strengths and limitations of the current study

Using data from the GBD 2021 study, this work provides a comprehensive assessment of the global, regional, and national burden of myocarditis in adolescents and young adults, and further evaluates improvement potential across 204 countries and territories through frontier analysis. Several limitations should be acknowledged. First, as with all GBD-based analyses, the accuracy of estimates depends on the availability and quality of underlying data sources. Heterogeneity in national reporting systems, cause-of-death coding practices, and health information infrastructure may introduce uncertainty, particularly in settings with sparse surveillance data. Although GBD partially accounts for these uncertainties through standardized modeling procedures and reports 95% uncertainty intervals derived from 1,000 posterior draws, absolute burden levels-especially in data-poor regions-should be interpreted conservatively. Second, myocarditis is clinically heterogeneous and can be difficult to diagnose. Differences in diagnostic capacity, access to cardiac biomarkers and imaging, and evolving case definitions across countries and over time may lead to under-ascertainment and misclassification, thereby affecting cross-national comparability and the interpretation of long-term trends, particularly in earlier years and low-resource settings. Importantly, under-ascertainment is expected to affect absolute levels more than relative comparisons (e.g., overall trend direction), and therefore results for low-SDI settings should be interpreted with caution. We revised the wording throughout the manuscript to clarify that the presented results are model-based estimates from GBD 2021 rather than directly observed surveillance counts, and that temporal patterns may partly reflect changes in diagnostic capacity and case ascertainment over time., and that temporal changes in incidence and mortality may partly reflect improvements in detection, diagnostic availability, and coding practices over time rather than true changes in underlying disease risk alone. To mitigate these concerns, we performed multiple robustness checks, including alternative joinpoint specifications for APC estimation, sensitivity analyses for frontier smoothing parameters, and prior sensitivity and retrospective validation for BAPC forecasts; the main conclusions remained consistent across specifications. Third, analyses of SDI patterns, climate-related context, and risk-factor attribution were conducted at the population level and may therefore be subject to ecological bias. SDI and other contextual indicators represent aggregate characteristics of countries or regions and may not apply to individuals with myocarditis; thus, group-level associations should not be interpreted as individual-level causal relationships. Future studies using individual-level clinical cohorts, standardized diagnostic criteria, and detailed exposure data are warranted to validate these findings and to better characterize patient-level determinants of myocarditis risk and outcomes in adolescents and young adults.

## Conclusion

Myocarditis among adolescents and young adults represents a pressing global public health concern, contributing to substantial healthcare demands and economic burden. Even if the worldwide incidence, mortality, and DALY rates are trending downward, the total number of incident cases, deaths, and DALYs is still increasing. These results highlight the pressing need for the adoption of focused, affordable preventative and control measures. Such efforts should aim not only to reduce the overall burden of myocarditis in this population but also to enhance family health outcomes and alleviate the broader socioeconomic impact on communities and health systems.

## Data Availability

The datasets presented in this study can be found in online repositories. The names of the repository/repositories and accession number(s) can be found in the article/Supplementary Material.
