# Supplementary material for: Global, regional and national burden of myocarditis in adolescents and young adults, 1990–2021: systematic analysis of the global burden of disease study 2021
Source: Front Cardiovasc Med. 2026 Mar 26;13:1623833. doi: 10.3389/fcvm.2026.1623833 (PMC13061660; doi:10.3389/fcvm.2026.1623833)
Supplement: Supplementary file 1 [file Datasheet1.zip › Supplementary Material 1/Frontier/DALYs/Figure S7-10.pdf]

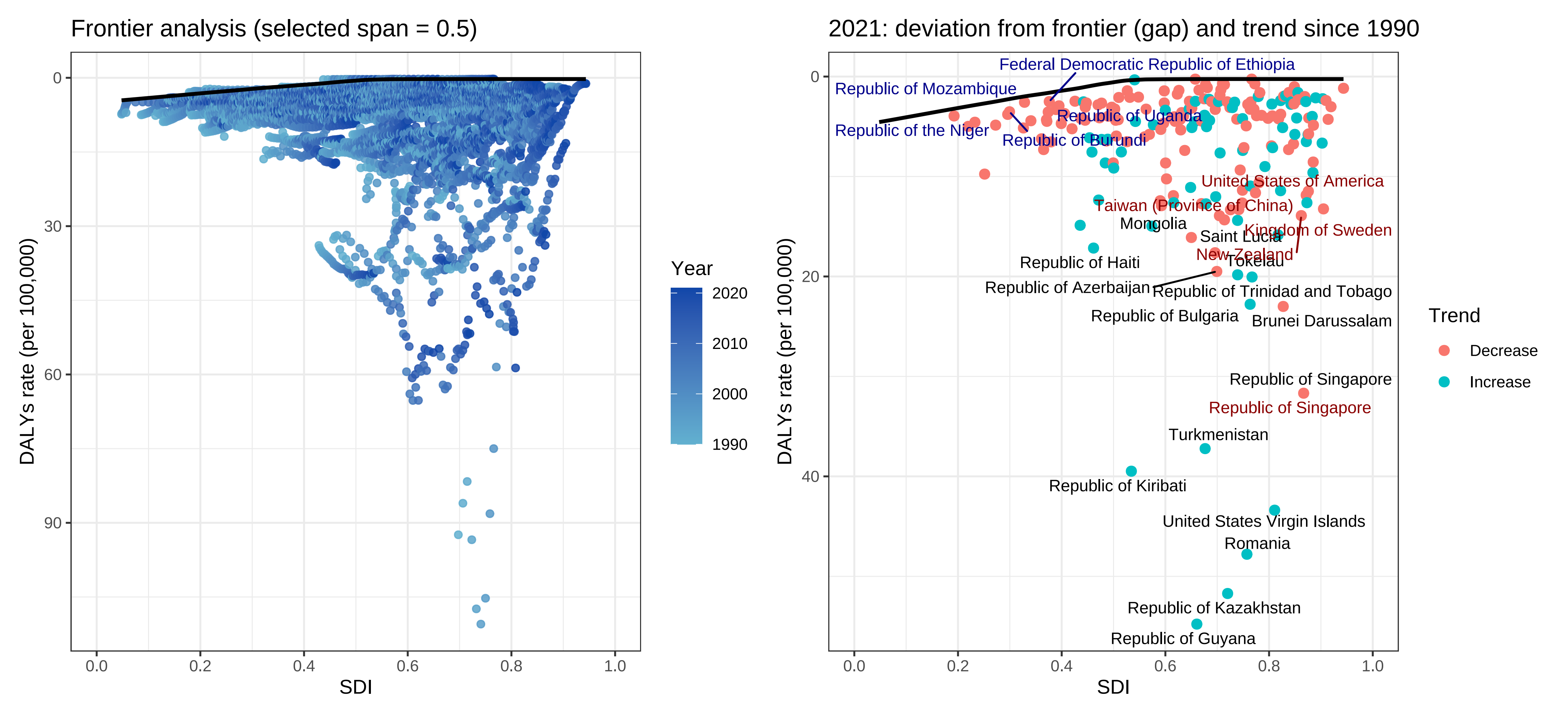

Frontier analysis (selected span = 0.5)
DALYs rate (per 100,000)
SDI
Year
2020
2010
2000
1990
2021: deviation from frontier (gap) and trend since 1990
Federal Democratic Republic of Ethiopia
Republic of Mozambique
Republic of Uganda
Republic of the Niger
Republic of Burundi
United States of America
Taiwan (Province of China)
Mongolia
Saint Lucia
Kingdom of Sweden
New Zealand
Tokelau
Republic of Haiti
Republic of Azerbaijan
Republic of Trinidad and Tobago
Republic of Bulgaria
Brunei Darussalam
Republic of Singapore
Republic of Singapore
Turkmenistan
Republic of Kiribati
United States Virgin Islands
Romania
Republic of Kazakhstan
Republic of Guyana
Trend
Decrease
Increase

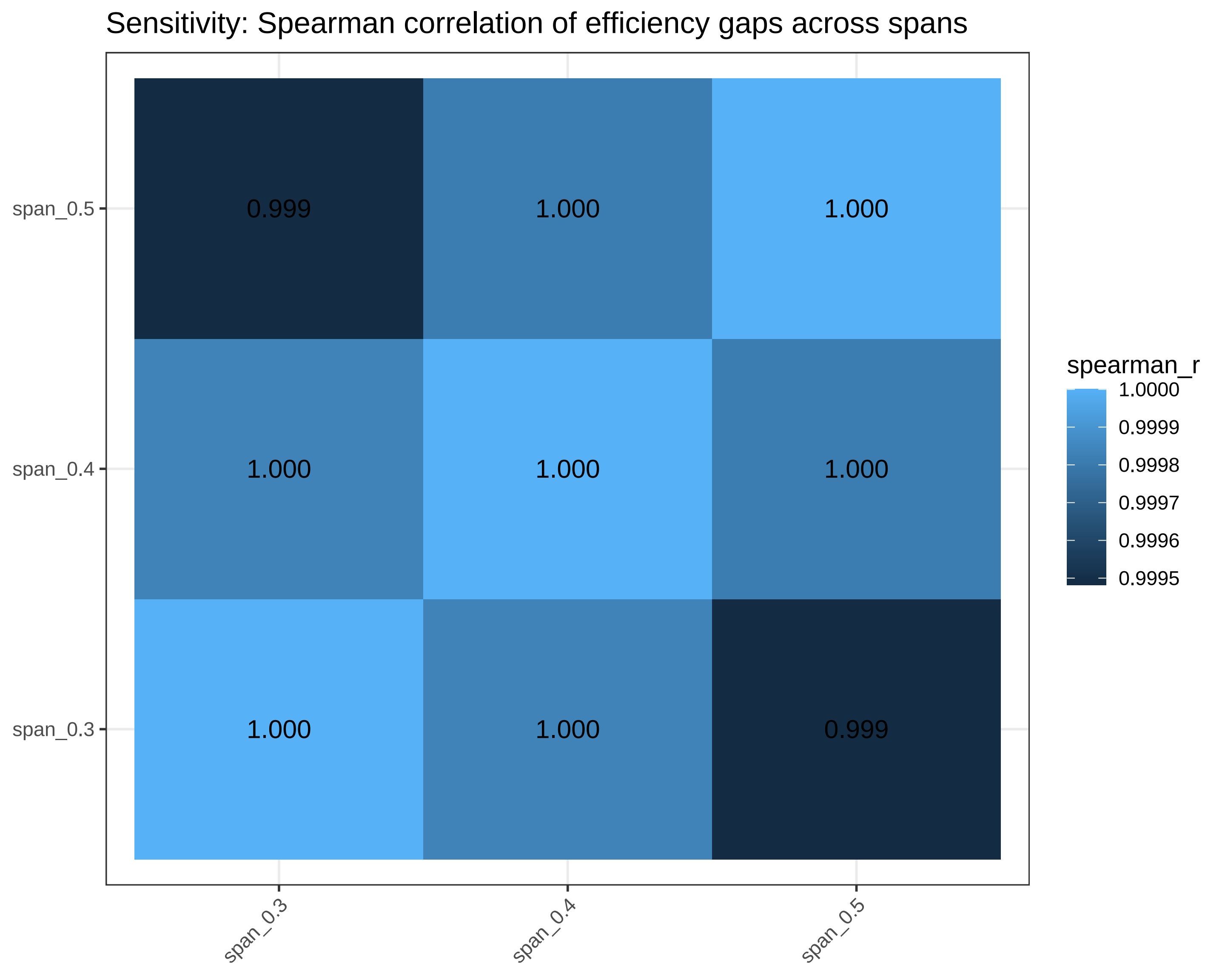

Sensitivity: Spearman correlation of efficiency gaps across spans
span_0.5
span_0.4
span_0.3
0.999
1.000
1.000
1.000
1.000
1.000
1.000
1.000
0.999
span_0.3
span_0.4
span_0.5
spearman_r
1.0000
0.9999
0.9998
0.9997
0.9996
0.9995

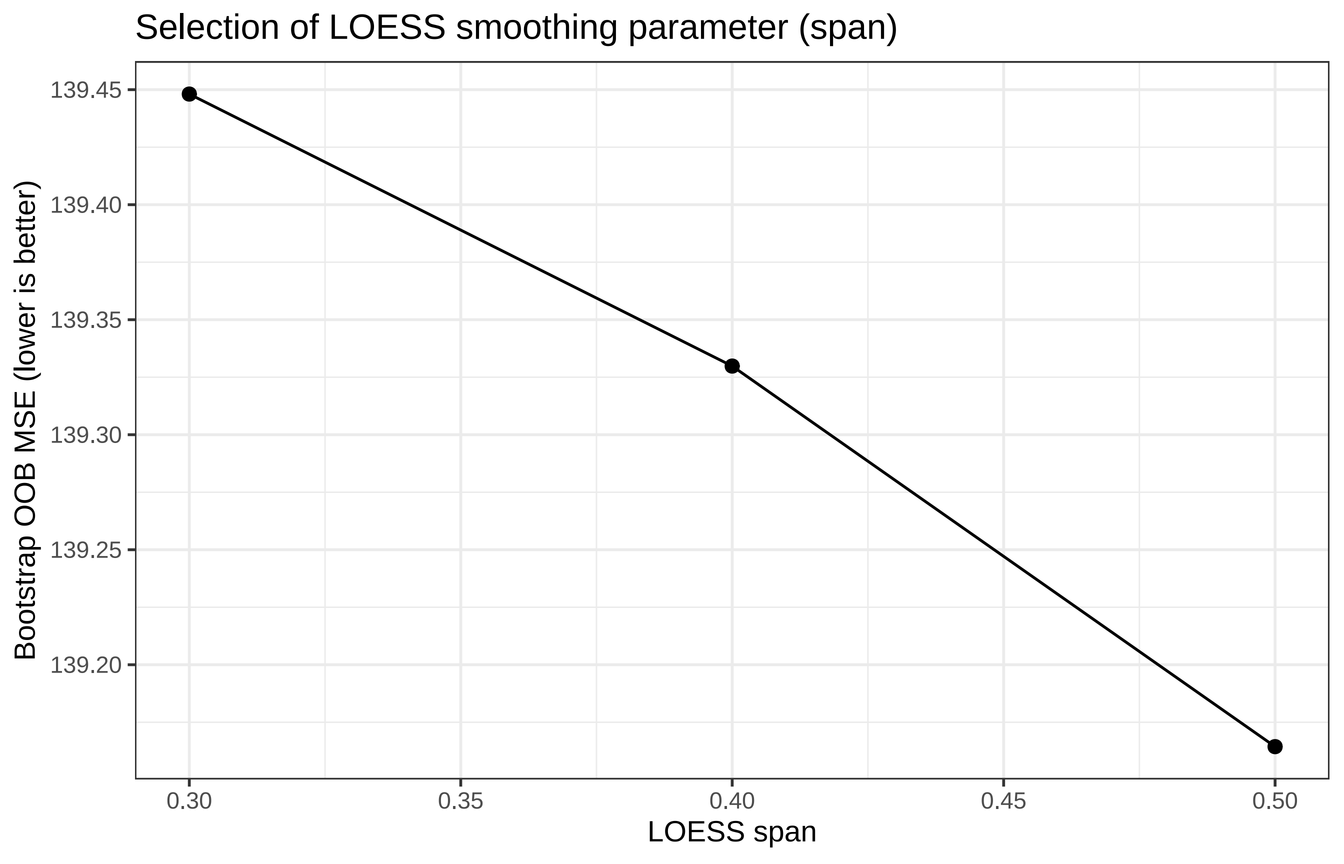
Selection of LOESS smoothing parameter (span)
Bootstrap OOB MSE (lower is better)
139.45
139.40
139.35
139.30
139.25
139.20
0.30
0.35
0.40
0.45
0.50
LOESS span

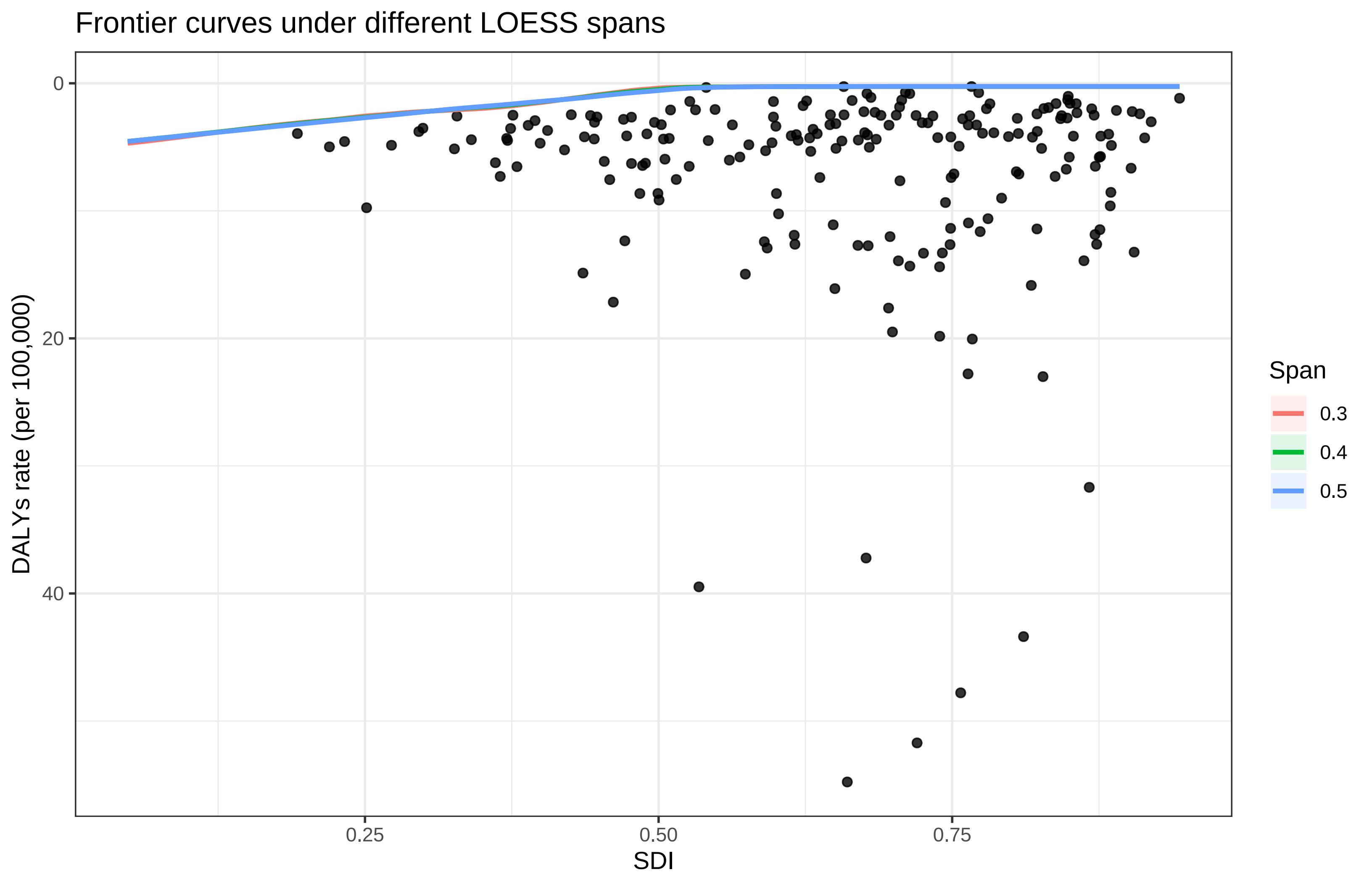

Frontier curves under different LOESS spans
DALYs rate (per 100,000)
0
20
40
0.25
0.50
0.75
SDI
Span
0.3
0.4
0.5
