# Supplementary figures and images for: Global, regional and national burden of myocarditis in adolescents and young adults, 1990–2021: systematic analysis of the global burden of disease study 2021
Source: Front Cardiovasc Med. 2026 Mar 26;13:1623833. doi: 10.3389/fcvm.2026.1623833 (PMC13061660; doi:10.3389/fcvm.2026.1623833)

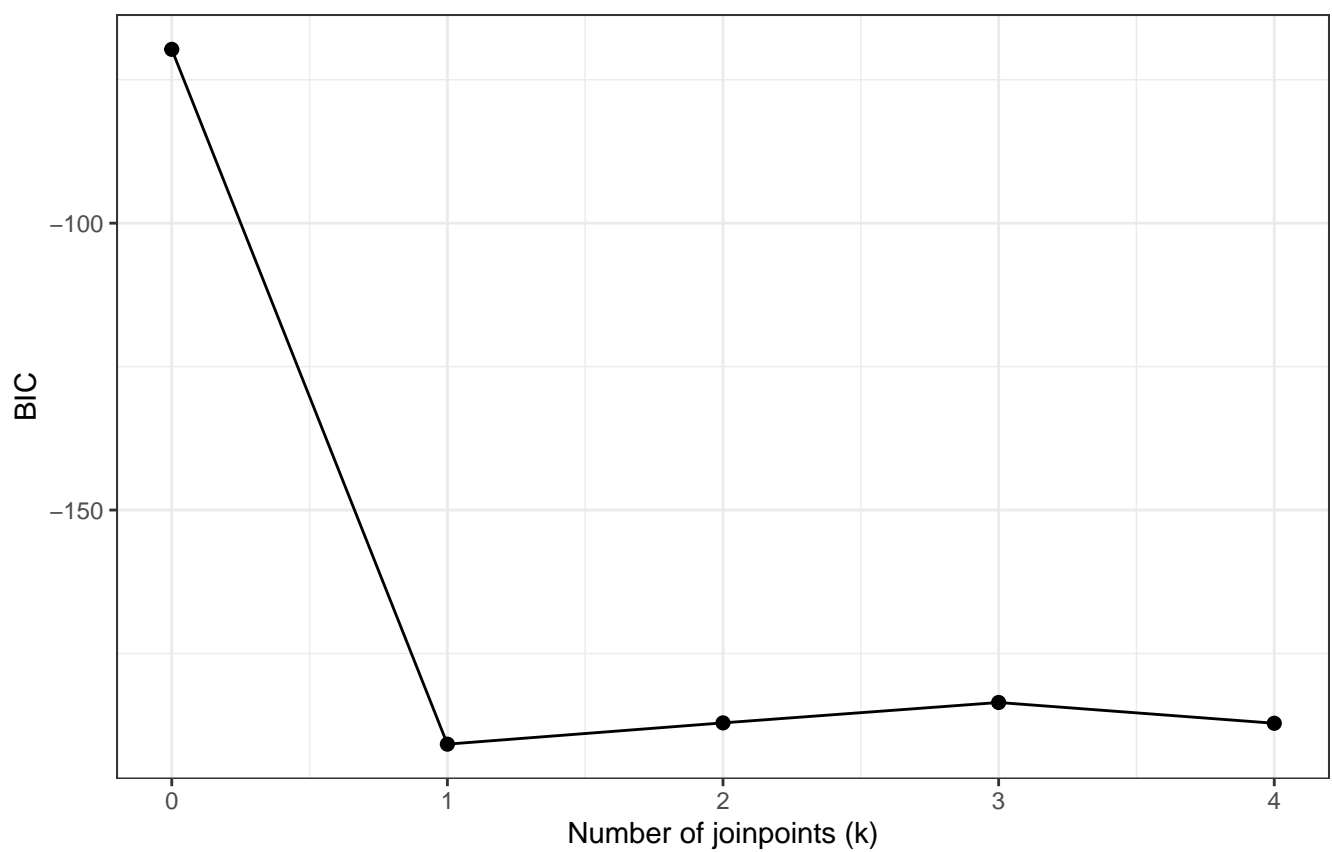

Residuals vs Fitted

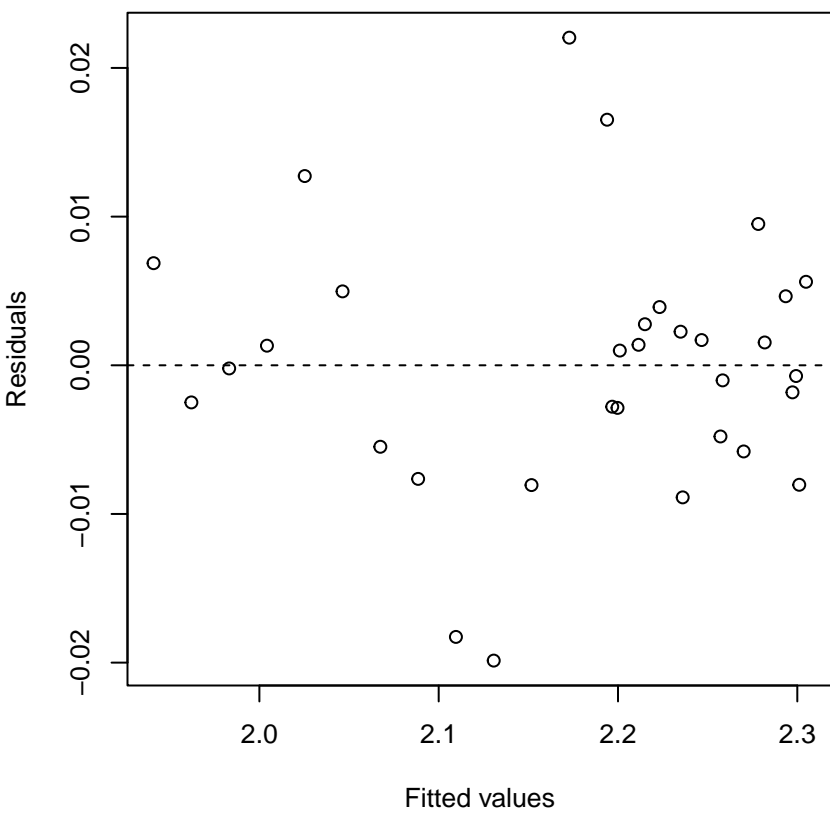

Normal Q-Q

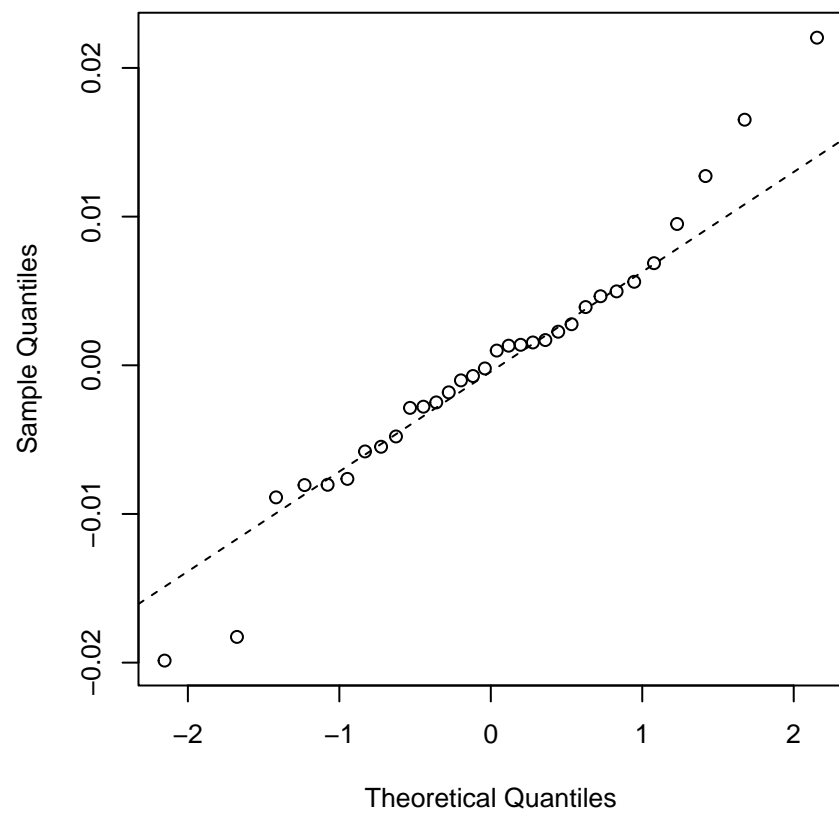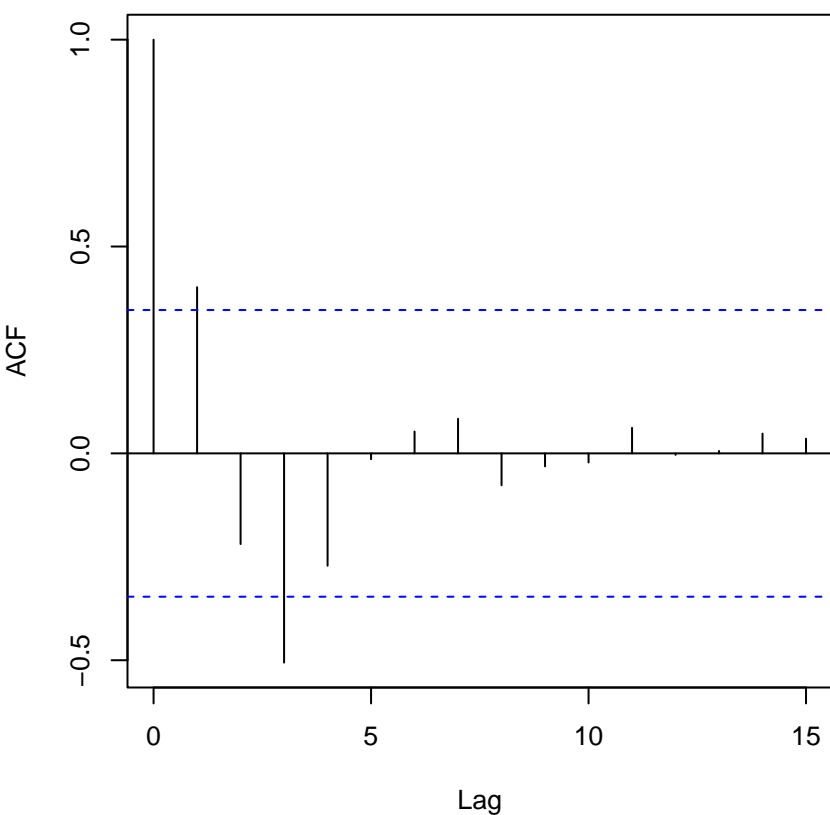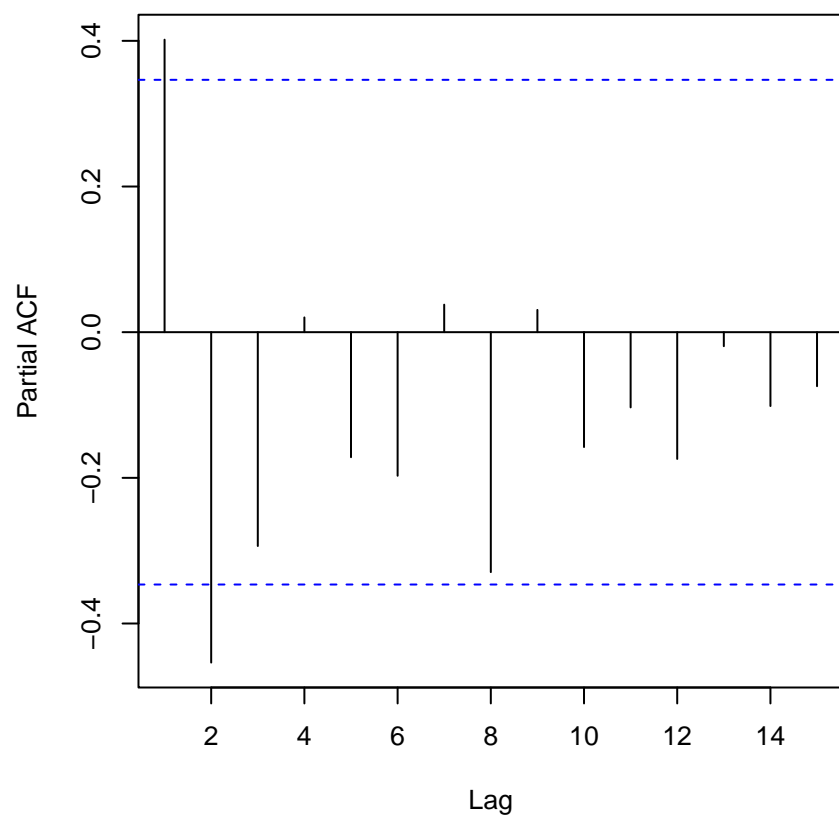

Supplement: Supplementary file 2 [file Datasheet2.zip › Supplementary Material 2/aapc/DALYs/Figure S11-12.pdf]

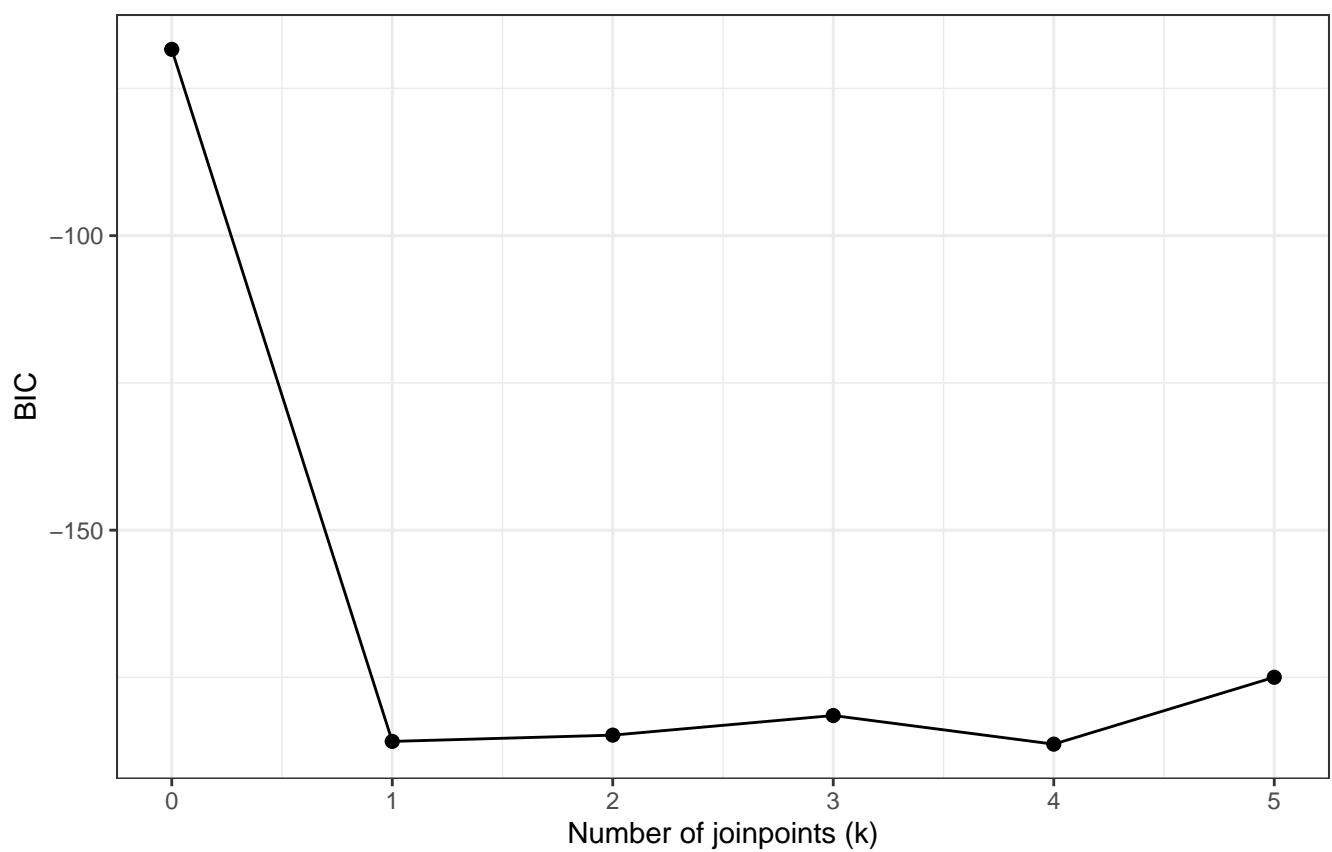

**Residuals vs Fitted**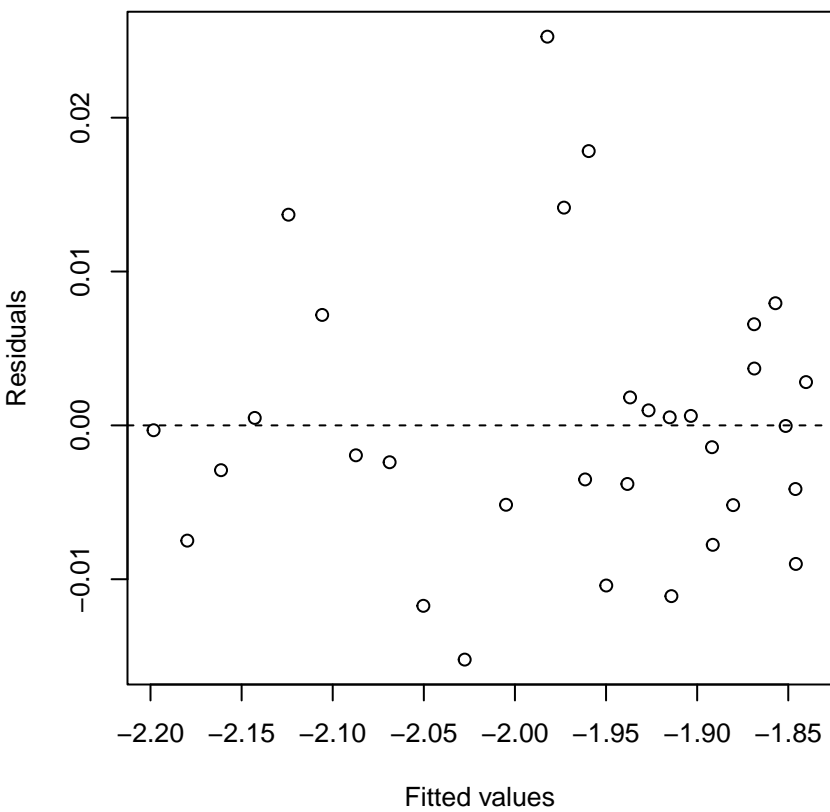**Normal Q-Q**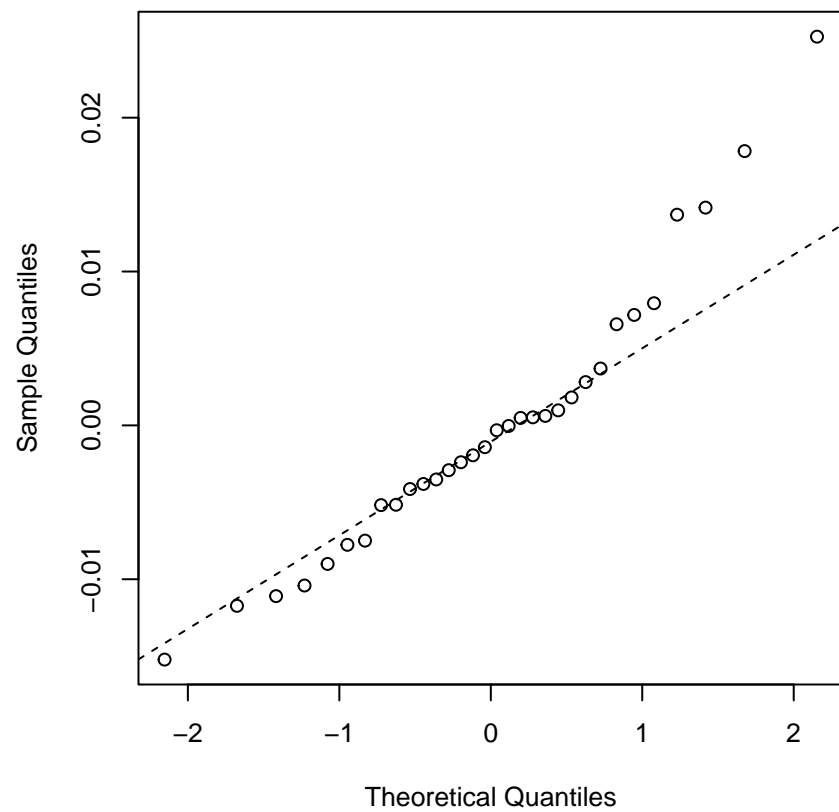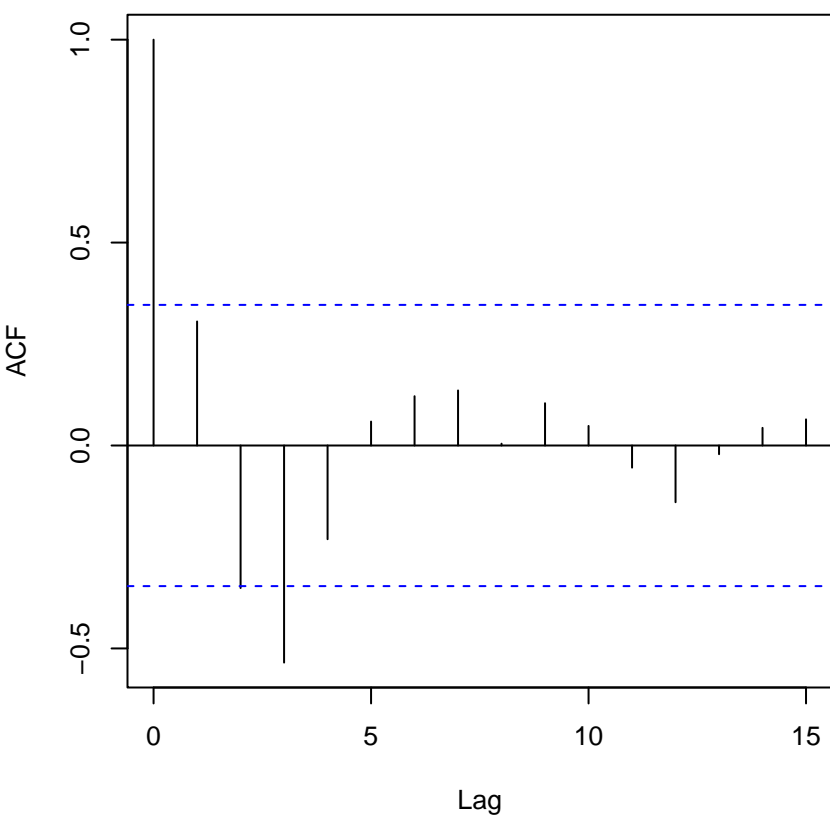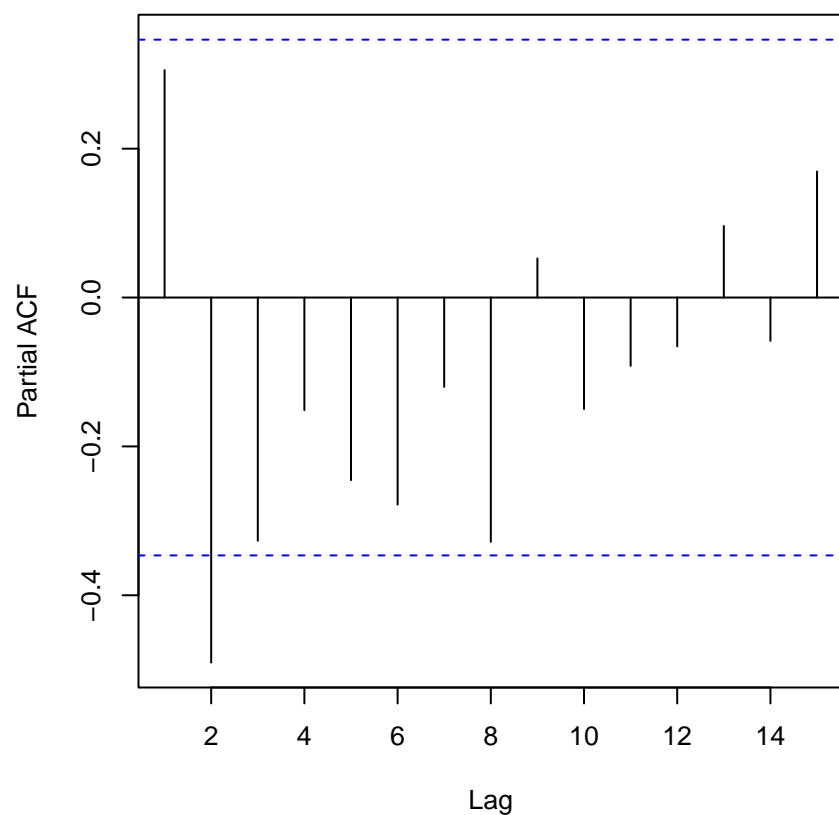

Supplement: Supplementary file 2 [file Datasheet2.zip › Supplementary Material 2/aapc/Deaths/Figure S13-14.pdf]

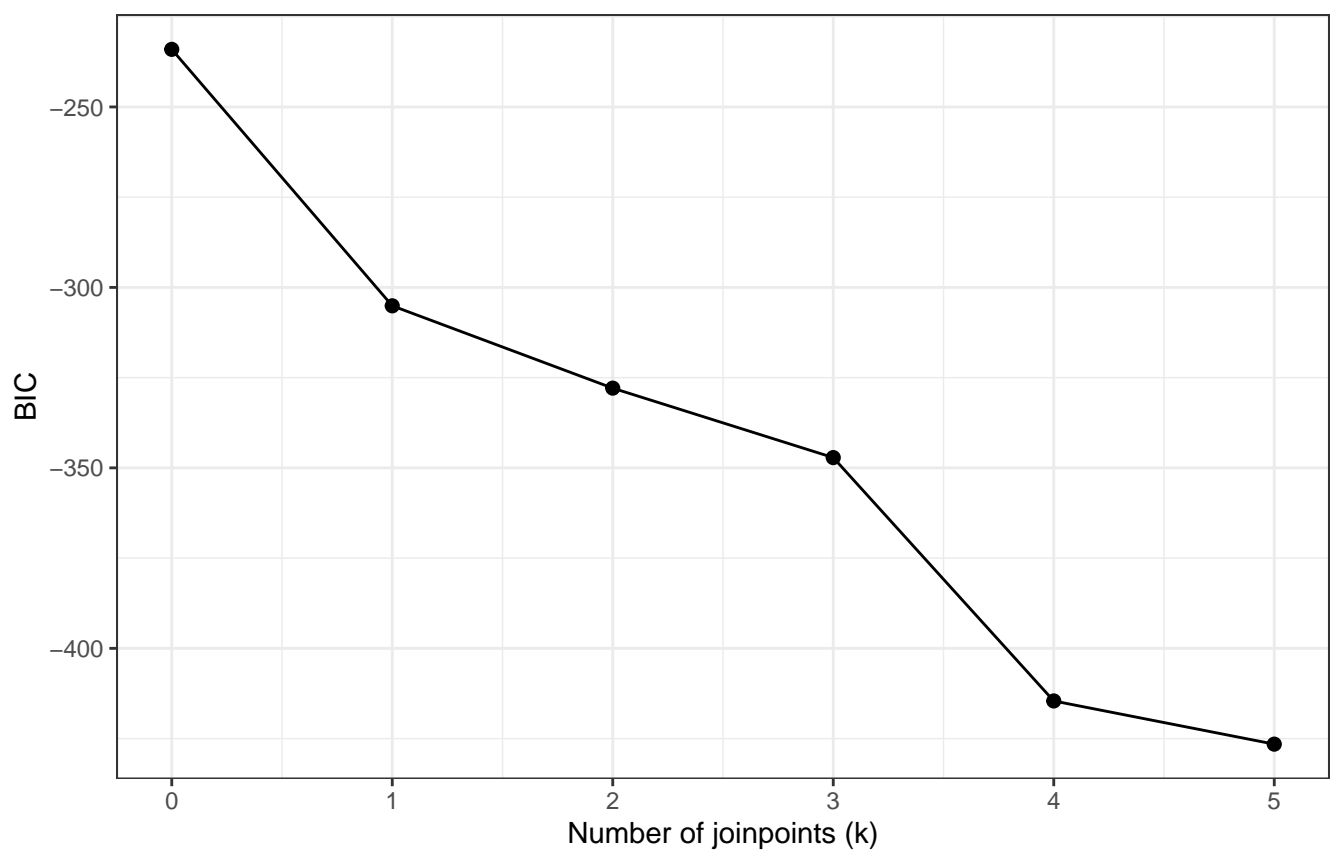

Residuals vs Fitted

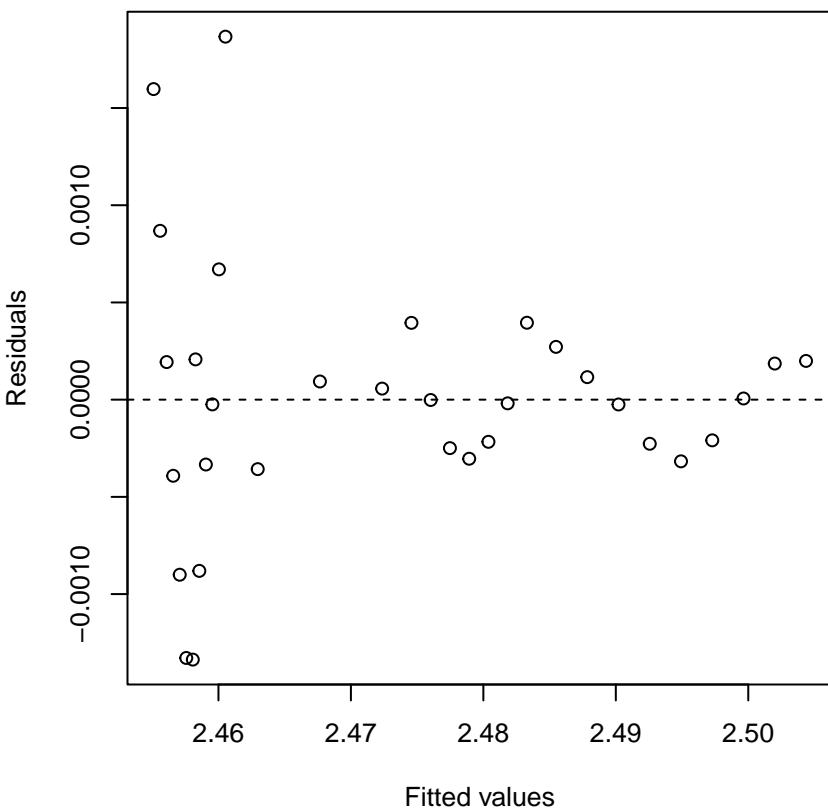

Normal Q-Q

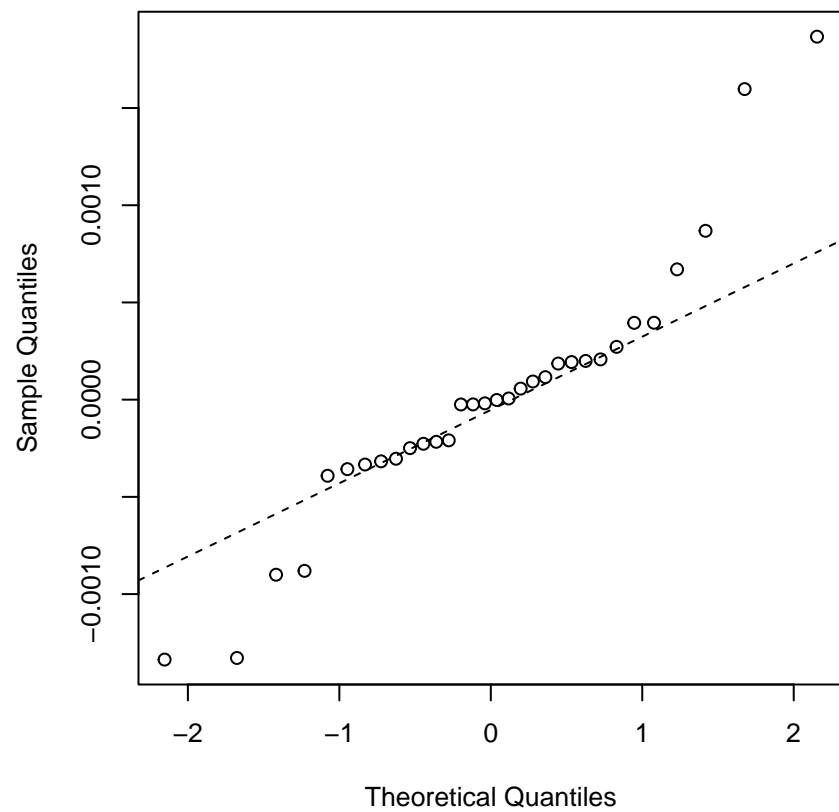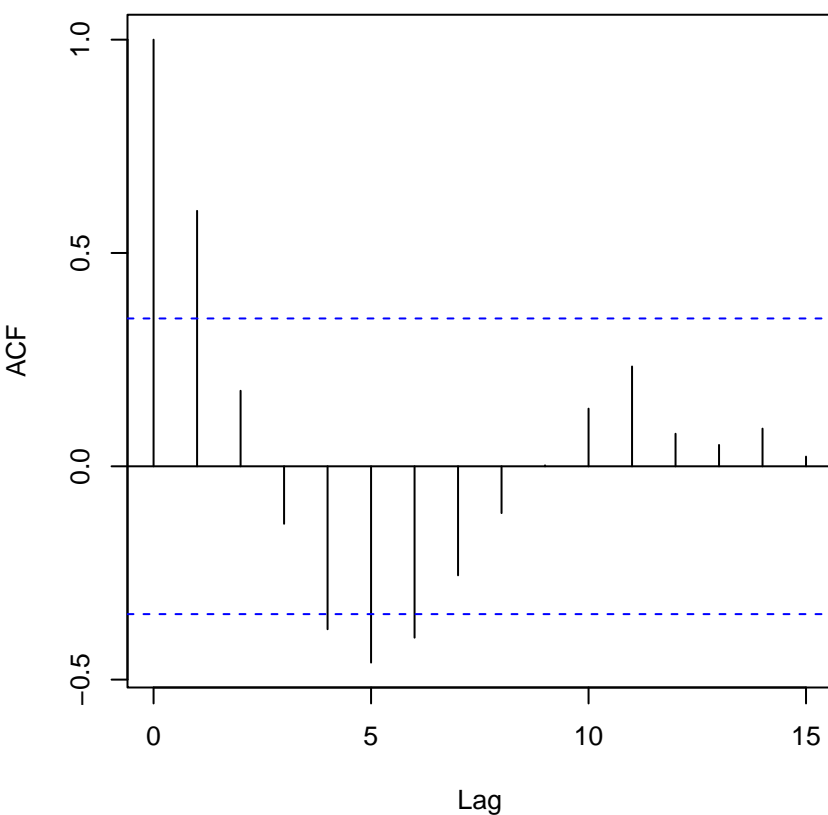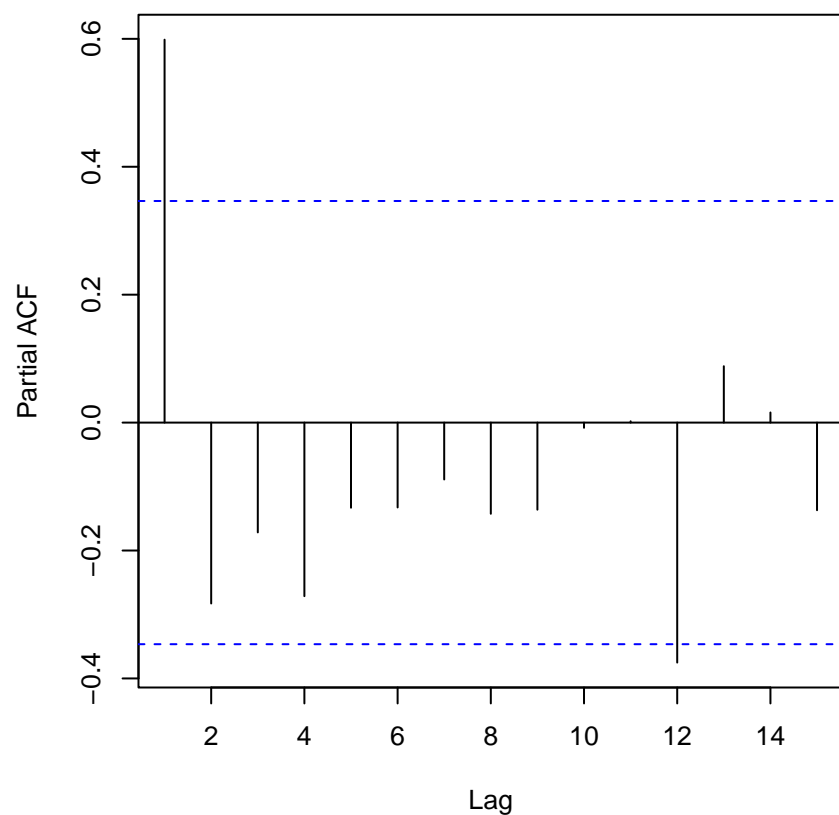

Supplement: Supplementary file 2 [file Datasheet2.zip › Supplementary Material 2/aapc/Incidence/Figure S15-16.pdf]

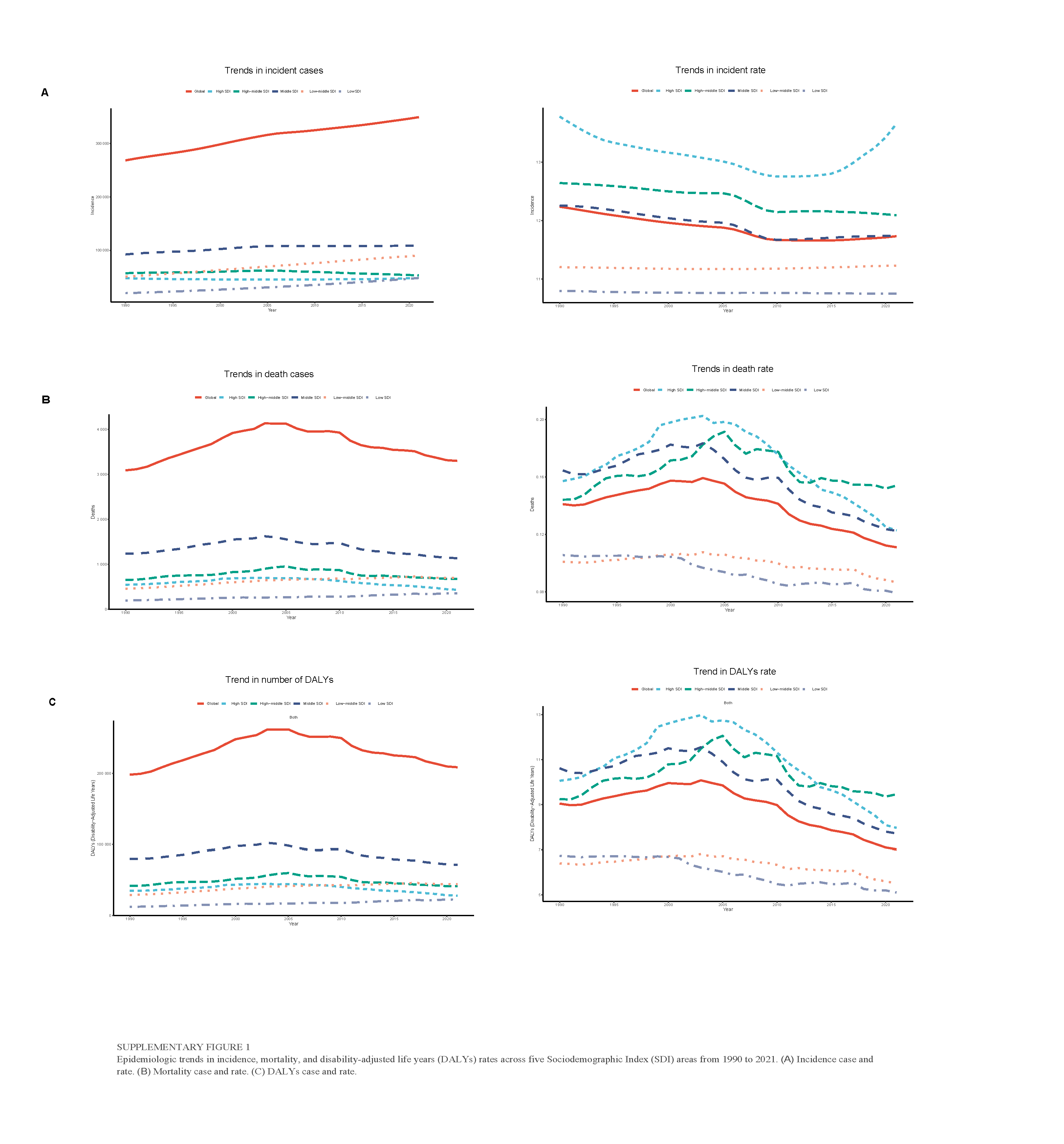

Supplement: Supplementary file 4 [file Image1.tif]

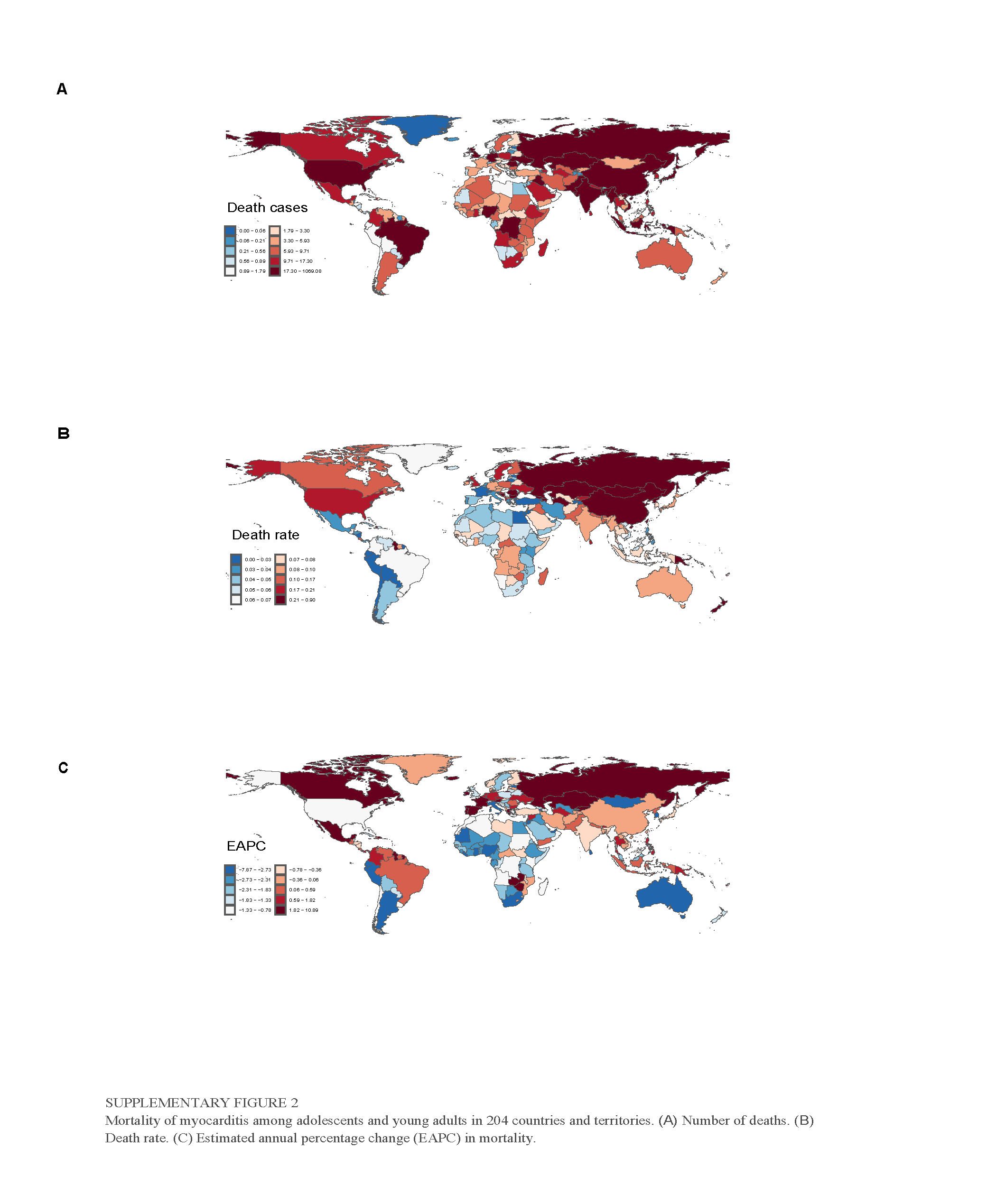

Supplement: Supplementary file 5 [file Image2.tif]

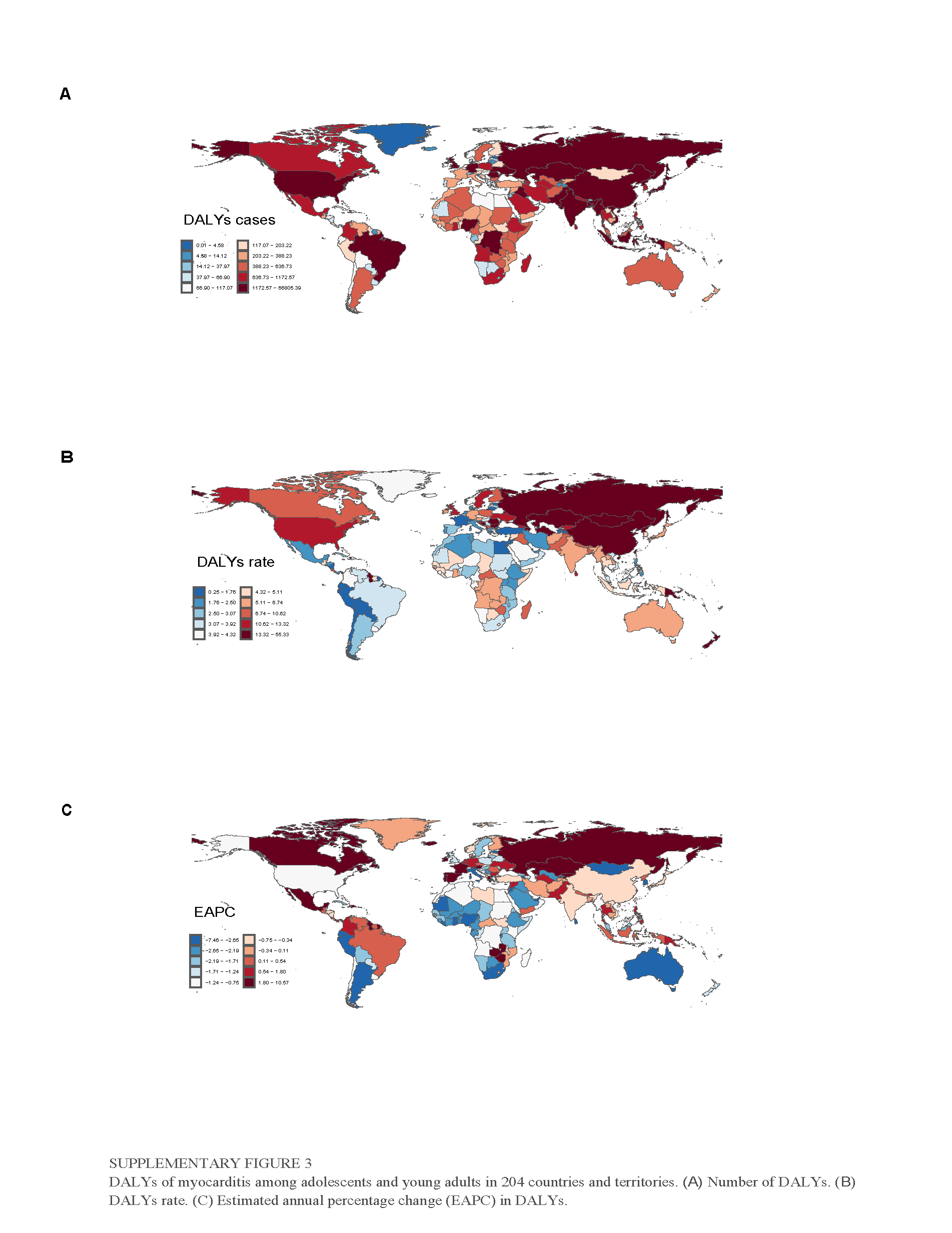

Supplement: Supplementary file 6 [file Image3.tif]

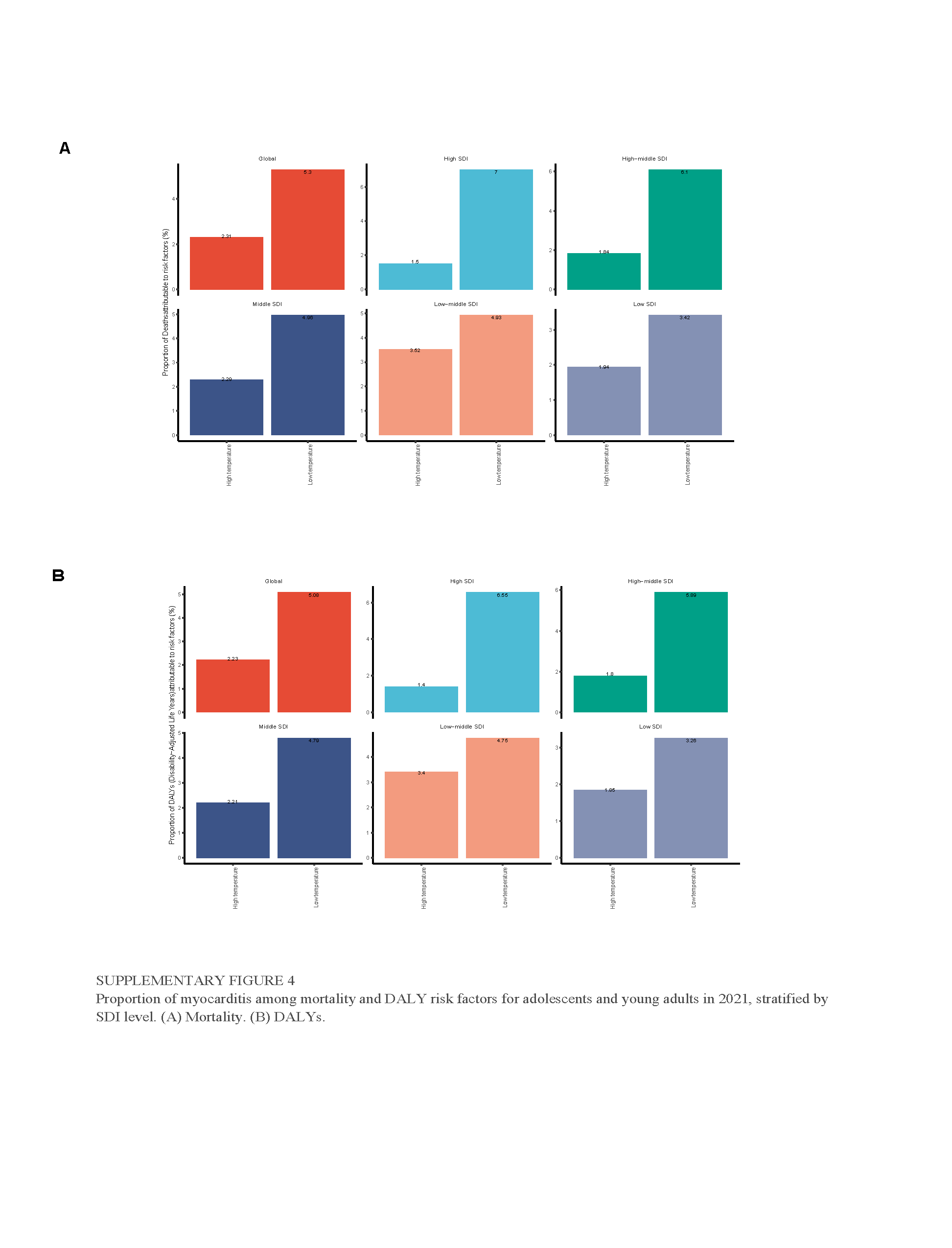

Supplement: Supplementary file 7 [file Image4.tif]

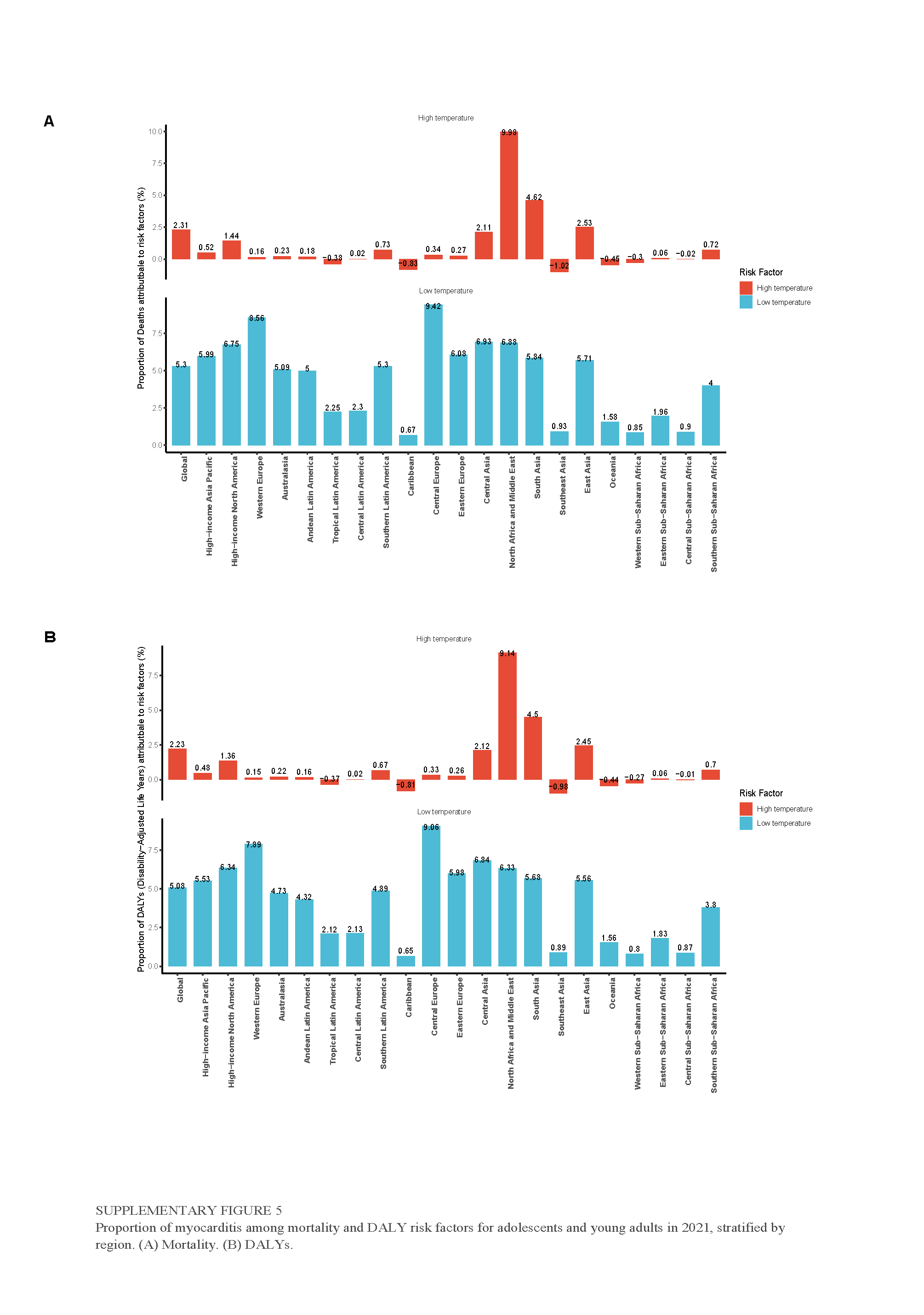

Supplement: Supplementary file 8 [file Image5.tif]

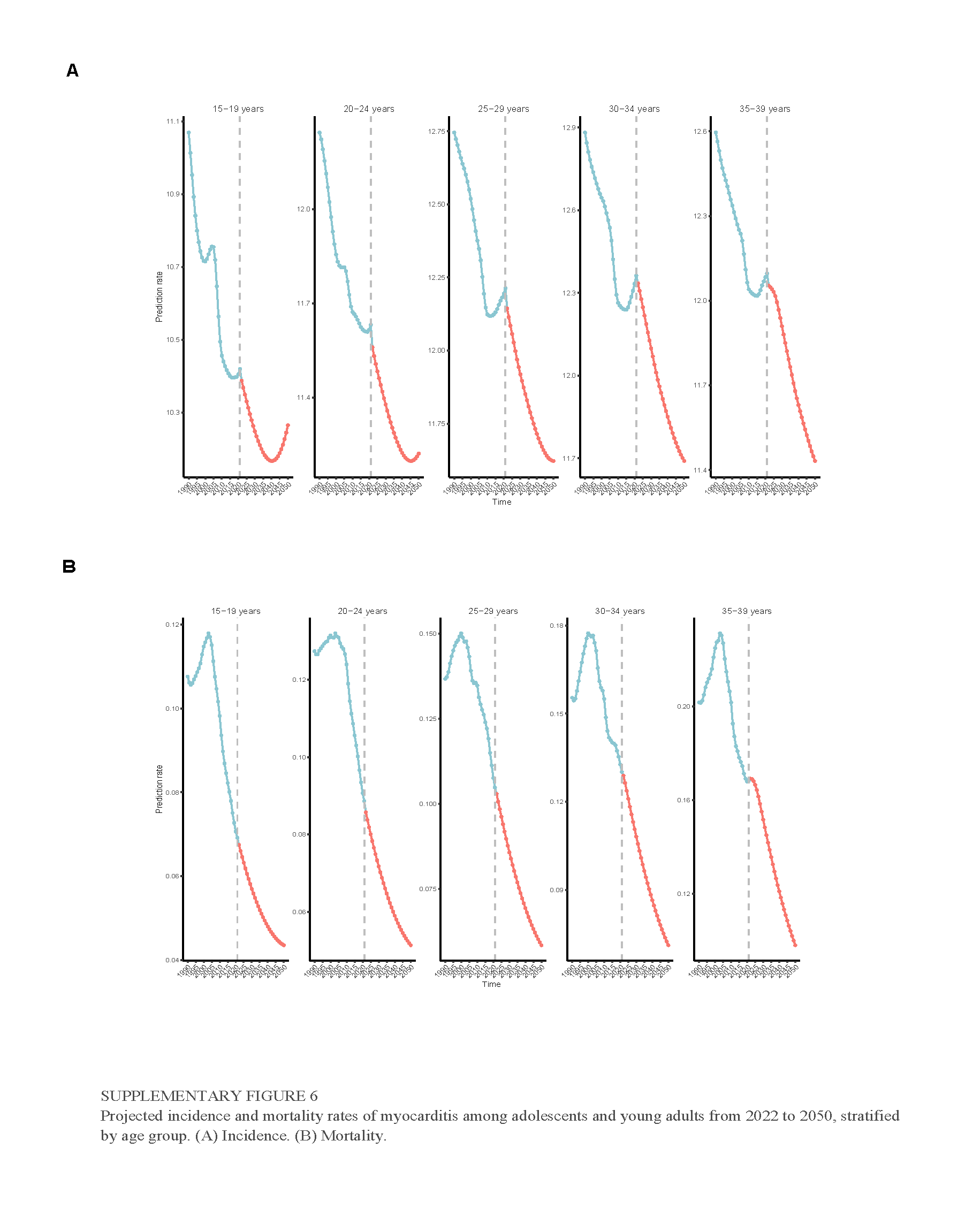

Supplement: Supplementary file 9 [file Image6.tif]
