# Supplementary material for: Global, regional and national burden of myocarditis in adolescents and young adults, 1990–2021: systematic analysis of the global burden of disease study 2021
Source: Front Cardiovasc Med. 2026 Mar 26;13:1623833. doi: 10.3389/fcvm.2026.1623833 (PMC13061660; doi:10.3389/fcvm.2026.1623833)
Supplement: Supplementary file 3 [file Datasheet3.zip › Supplementary Material 3/BAPC/Myocarditis_DALYs_Both_loc1_single_pdfs/Figure S17-20.pdf]

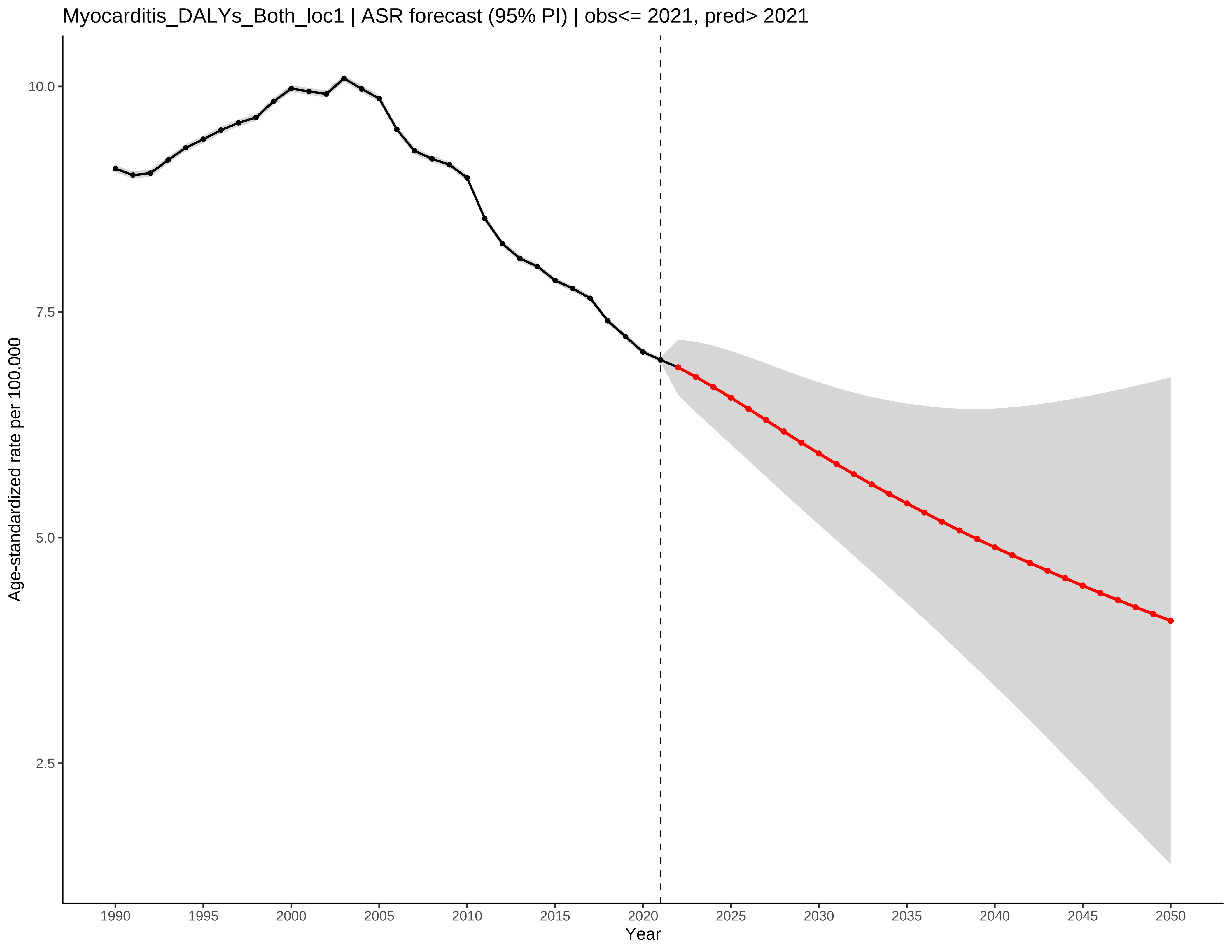

Myocarditis_DALYs_Both_loc1 | ASR forecast (95% PI) | obs<= 2021, pred> 2021
Age-standardized rate per 100,000
10.0
7.5
5.0
2.5
1990
1995
2000
2005
2010
2015
2020
2025
2030
2035
2040
2045
2050
Year

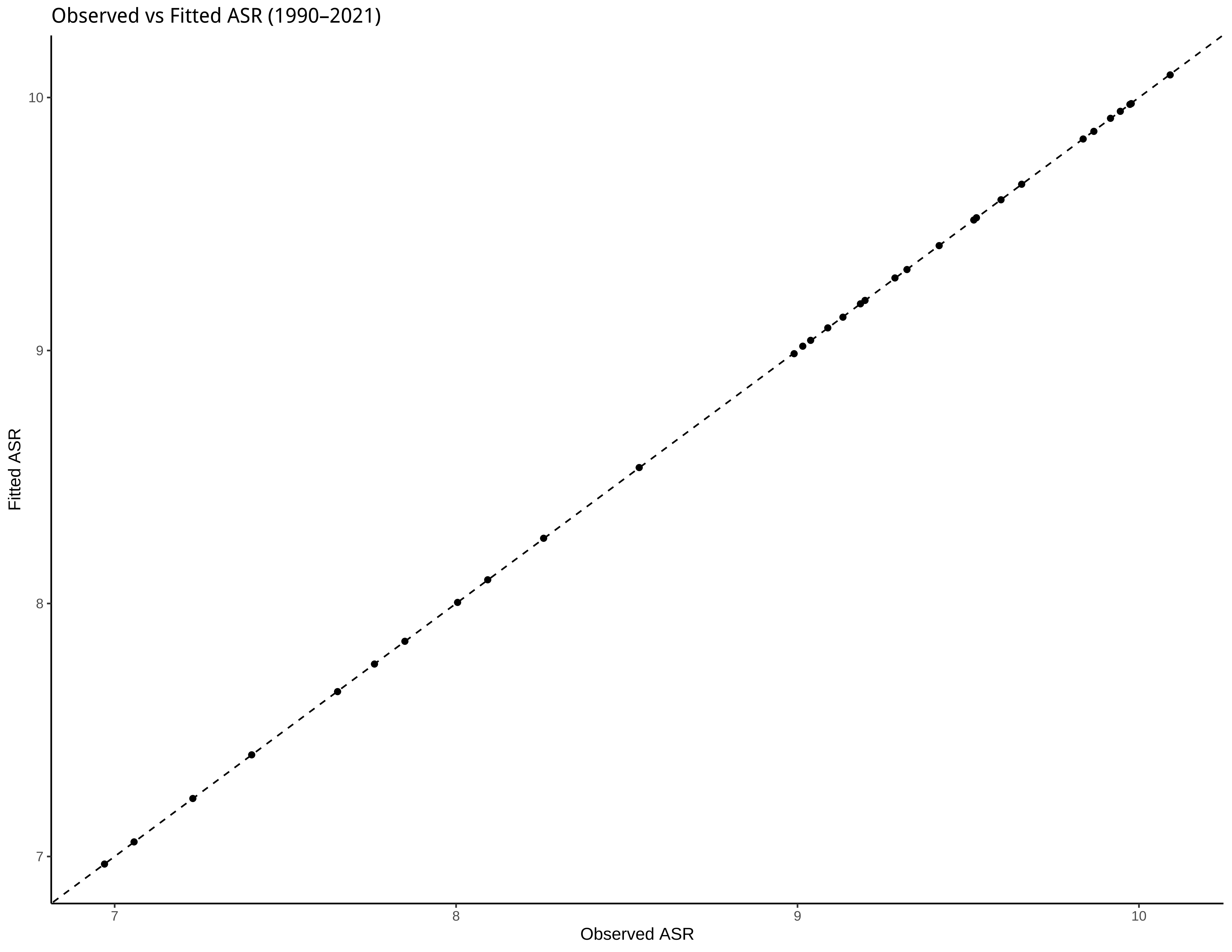
Observed vs Fitted ASR (1990–2021)
Fitted ASR
Observed ASR
10
9
8
7
7
8
9
10

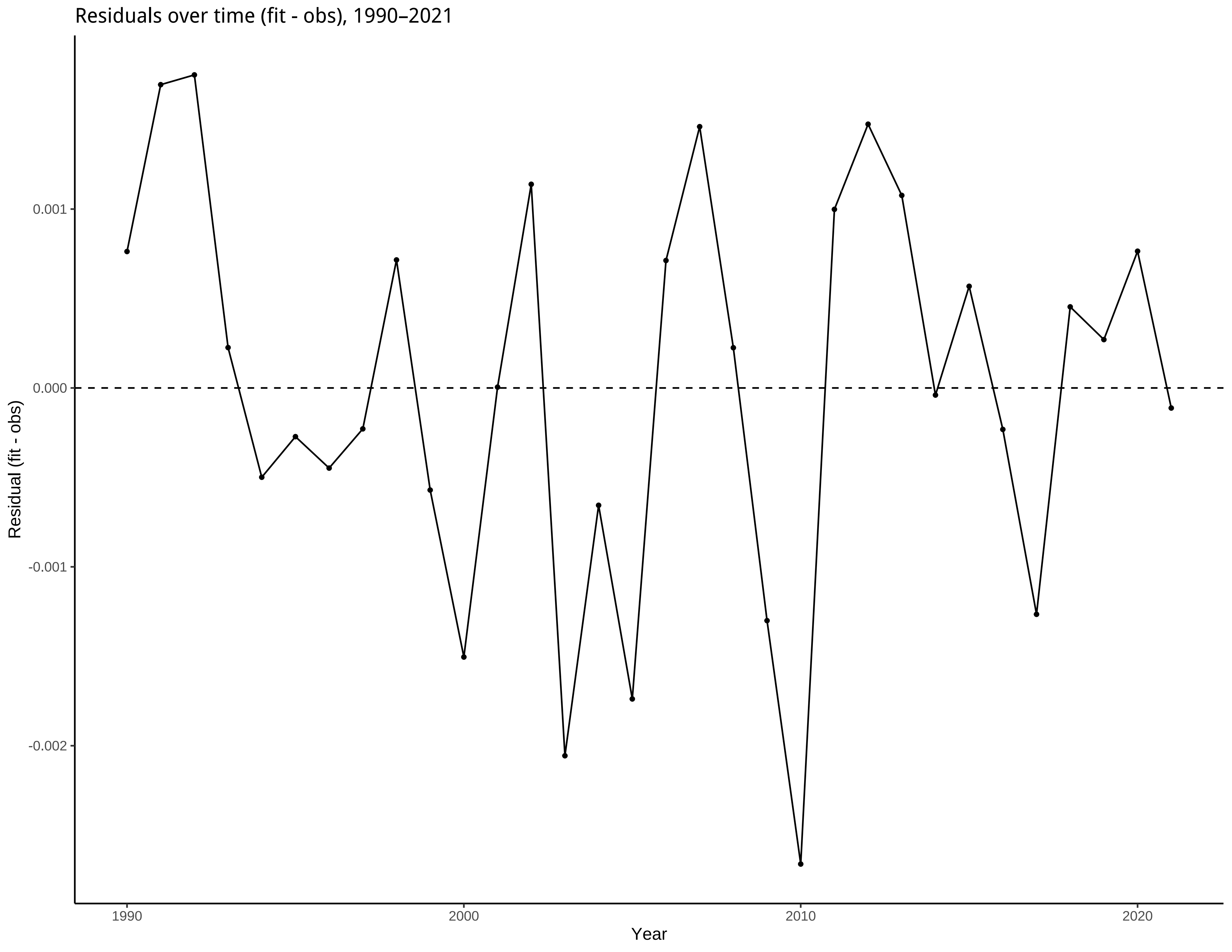

Residuals over time (fit - obs), 1990–2021
0.001
0.000
-0.001
-0.002
Residual (fit - obs)
1990
2000
2010
2020
Year

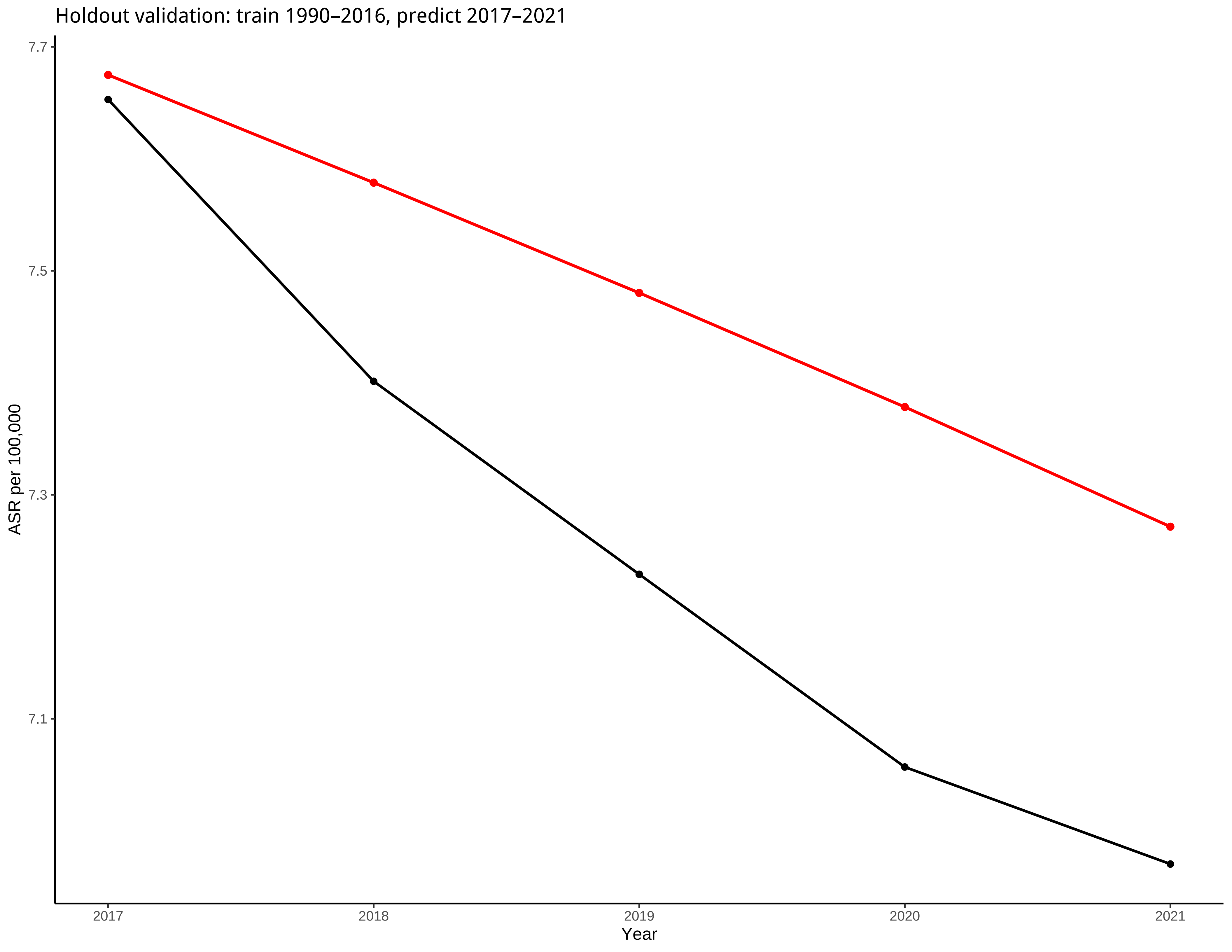
Holdout validation: train 1990–2016, predict 2017–2021
ASR per 100,000
7.7
7.5
7.3
7.1
2017
2018
2019
2020
2021
Year

Model specification (as implemented in code):
- Age: RW2, prior log-gamma(1, 0.00005)
- Period: RW1, prior log-gamma(1, 0.00005)
- Cohort: RW2, prior log-gamma(1, 0.00005)
- Overdispersion: IID random effect, prior log-gamma(1, 0.005)

Run info:
Full fit: fallback_used = FALSE; message: Custom prior success.
Holdout fit: fallback_used = FALSE; message: Custom prior success.

Goodness-of-fit (1990–2021):
MAE = 0.0008713; RMSE = 0.001088; MAPE(%) = 0.009709; cor = 1

Holdout (2017–2021):
MAE = 0.2147; RMSE = 0.2405; MAPE(%) = 3.008; cor = 0.9827
