# Supplementary material for: Global, regional and national burden of myocarditis in adolescents and young adults, 1990–2021: systematic analysis of the global burden of disease study 2021
Source: Front Cardiovasc Med. 2026 Mar 26;13:1623833. doi: 10.3389/fcvm.2026.1623833 (PMC13061660; doi:10.3389/fcvm.2026.1623833)
Supplement: Supplementary file 3 [file Datasheet3.zip › Supplementary Material 3/BAPC/Myocarditis_Incidence_Both_loc1_single_pdfs/Figure S25-28.pdf]

Myocarditis_Incidence_Both_loc1 | ASR forecast (95% PI) | obs<= 2021, pred> 2021

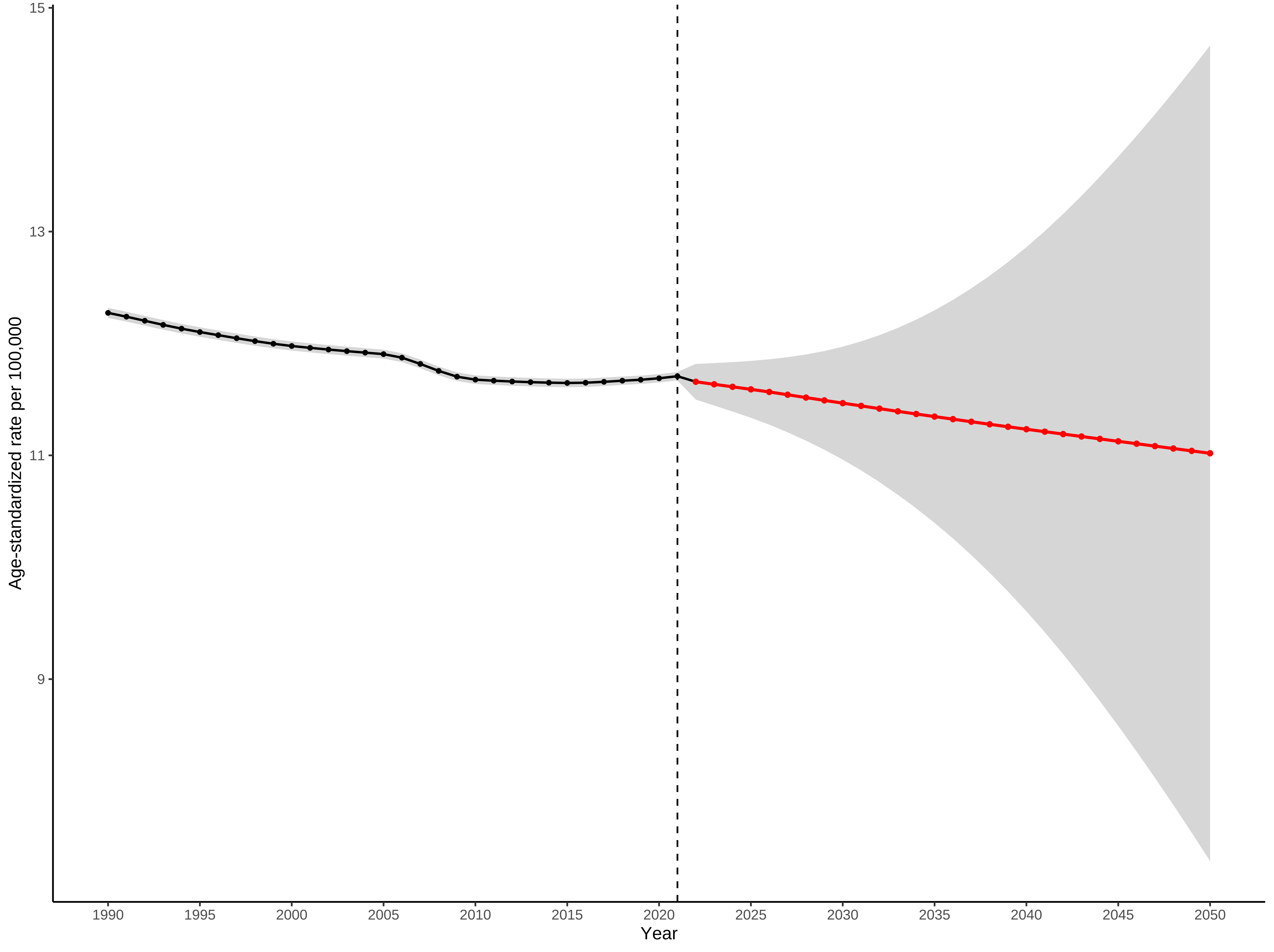

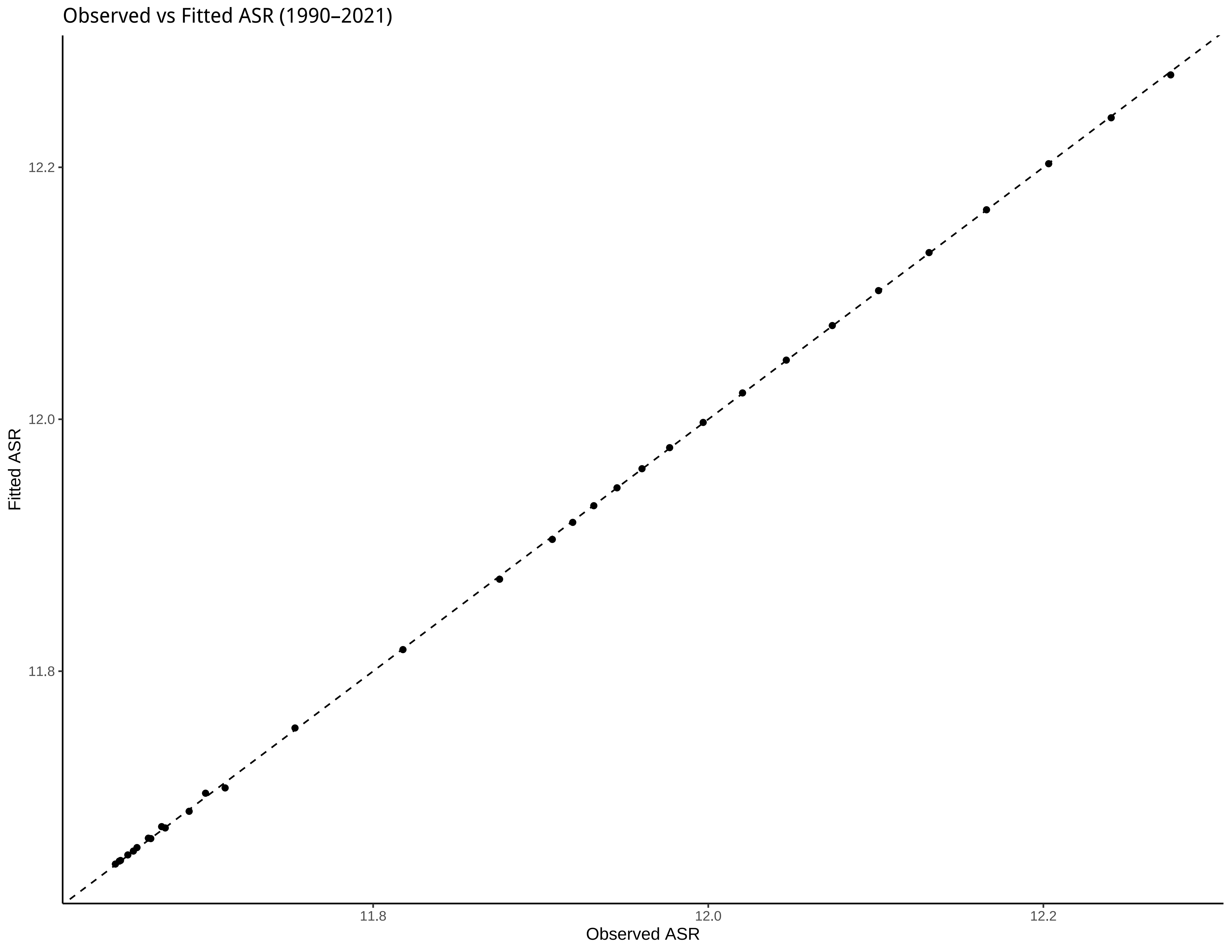
Observed vs Fitted ASR (1990–2021)
Fitted ASR
12.2
12.0
11.8
11.8
12.0
12.2
Observed ASR

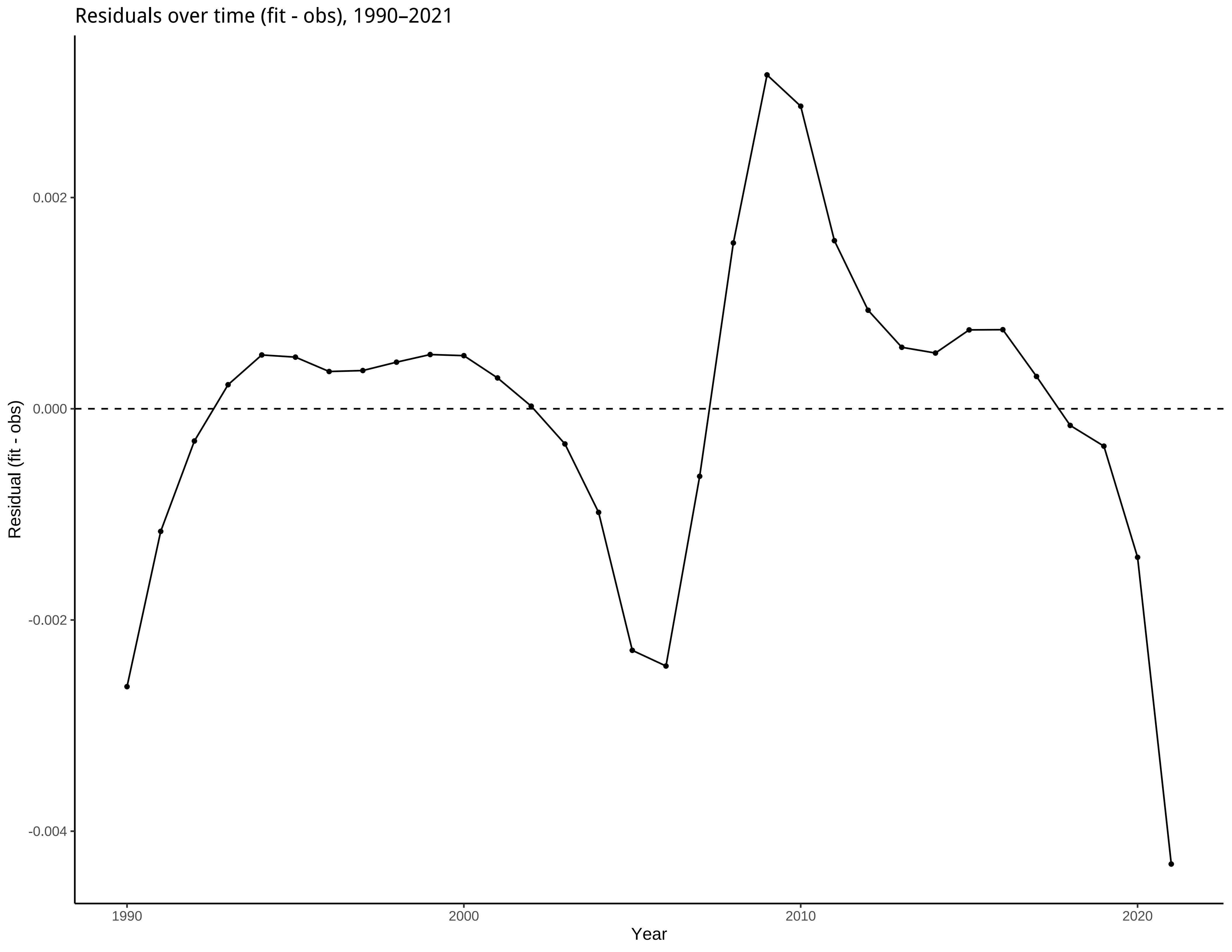
Residuals over time (fit - obs), 1990–2021
0.002
0.000
-0.002
-0.004
Residual (fit - obs)
1990
2000
2010
2020
Year

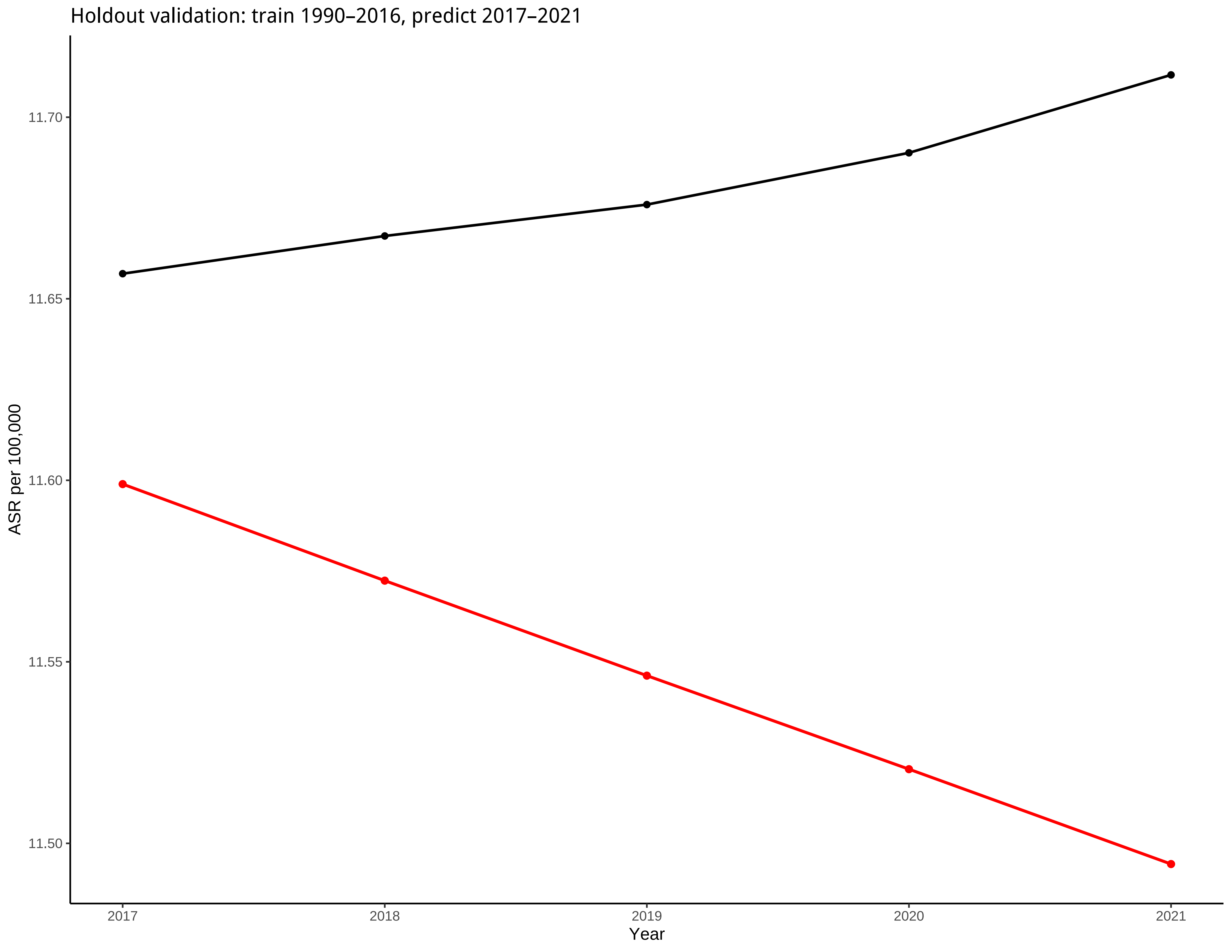
Holdout validation: train 1990–2016, predict 2017–2021
ASR per 100,000
11.70
11.65
11.60
11.55
11.50
2017
2018
2019
2020
2021
Year

Model specification (as implemented in code):
- Age: RW2, prior log-gamma(1, 0.00005)
- Period: RW1, prior log-gamma(1, 0.00005)
- Cohort: RW2, prior log-gamma(1, 0.00005)
- Overdispersion: IID random effect, prior log-gamma(1, 0.005)

Run info:
Full fit: fallback_used = FALSE; message: Custom prior success.
Holdout fit: fallback_used = FALSE; message: Custom prior success.

Goodness-of-fit (1990–2021):
MAE = 0.001055; RMSE = 0.001471; MAPE(%) = 0.008912; cor = 1

Holdout (2017–2021):
MAE = 0.134; RMSE = 0.1451; MAPE(%) = 1.146; cor = -0.9818
